# 4C-seq characterization of *Drosophila* BEAF binding regions provides evidence for highly variable long-distance interactions between active chromatin

**DOI:** 10.1371/journal.pone.0203843

**Published:** 2018-09-24

**Authors:** Shraddha Shrestha, Dong-Ha Oh, J. Keller McKowen, Maheshi Dassanayake, Craig M. Hart

**Affiliations:** Department of Biological Sciences, Louisiana State University, Baton Rouge, Louisiana, United States of America; Emory University Rollins School of Public Health, UNITED STATES

## Abstract

Chromatin organization is crucial for nuclear functions such as gene regulation, DNA replication and DNA repair. Insulator binding proteins, such as the *Drosophila* Boundary Element-Associated Factor (BEAF), are involved in chromatin organization. To further understand the role of BEAF, we detected *cis-* and *trans-*interaction partners of four BEAF binding regions (viewpoints) using 4C (circular chromosome conformation capture) and analyzed their association with different genomic features. Previous genome-wide mapping found that BEAF usually binds near transcription start sites, often of housekeeping genes, so our viewpoints were selected to reflect this. Our 4C data show the interaction partners of our viewpoints are highly variable and generally enriched for active chromatin marks. The most consistent association was with housekeeping genes, a feature in common with our viewpoints. Fluorescence in situ hybridization indicated that the long-distance interactions occur even in the absence of BEAF. These data are most consistent with a model in which BEAF is redundant with other factors found at active promoters. Our results point to principles of long-distance interactions made by active chromatin, supporting a previously proposed model in which condensed chromatin is sticky and associates into topologically associating domains (TADs) separated by active chromatin. We propose that the highly variable long-distance interactions we detect are driven by redundant factors that open chromatin to promote transcription, combined with active chromatin filling spaces between TADs while packing of TADs relative to each other varies from cell to cell.

## Introduction

Multiple studies have shown the importance of chromatin architecture for proper gene regulation (for example, [1–3]). The development of 3C (chromosome conformation capture) and 3C-related methods such as 4C, 5C, Hi-C and ChIA-PET, have helped us understand the complexity of genome organization in three-dimensional space [4, 5]. For example, such studies have found that chromosomes are organized into topologically associating domains (TADs). Insulators, or boundary elements, are a class of architectural elements necessary to maintain a healthy functioning genome [6–8]. They are defined as specialized protein-bound DNA elements known to play an important role in chromatin organization and gene regulation by influencing intra- and inter-chromosomal interactions. There are two classical assays of insulator function. One is the protection of transgenes bracketed by insulators from chromosomal position effects [9, 10]. The other is the blocking of enhancer-promoter communication by an intervening insulator, for instance by insertion of an insulator-containing *gypsy* retrotransposon [11, 12]. These functions are thought to be important for protecting genes from chromosomal position effects and preventing promiscuous interactions between long-distance regulatory elements and promoters, and are presumably related to their role in genome organization.

Insulators need insulator binding proteins (IBPs) to function. Of the many IBPs found in *Drosophila*, Boundary Element-Associated Factor-32 (BEAF) is our protein of interest [13]. There are two 32 kDa BEAF proteins (BEAF-32A and BEAF-32B, hereafter referred to as 32A and 32B) made from alternative promoters of the same gene [14]. The two isomers of BEAF differ in their N-termini by 80 amino acids which contain single DNA-binding BED zinc fingers, and interact via a BESS domain located in their C-termini [15–17]. The number of BEAF subunits in a BESS-mediated complex is unknown, and might be variable.

BEAF was discovered based on its binding to the scs’ insulator [13], one of the first insulators to be described together with scs as special chromatin structures bracketing the *87A hsp70* heat shock locus [9, 11, 18]. There are two BEAF binding sites in scs’, both with clusters of 3 CGATA motifs. The BED finger of 32B binds this motif [14], and 32B is essential while 32A is not [19]. The CGATA motifs are important for scs’ insulator function, and clusters are found in other sequences that BEAF binds to that have been shown to have insulator activity [10, 20]. This led to the model that BEAF binds 3 or more CGATA motifs clustered in a 100 bp region. A bioinformatics approach found that around 1700 clusters matching this model also have a second cluster of 2 or more CGATA motifs in a 50 bp region located 200 bp to 1 kb from the first cluster, an arrangement termed a dual-core [21]. Genome-wide mapping found 1800 to 3000 BEAF peaks and confirmed that CGATA clusters are frequent in BEAF binding regions [22–25]. However, the relative orientations and spacing of CGATA motifs in clusters is highly variable and many peak regions have only one or no CGATA motifs. Electrophoretic mobility shift assays found that CGATA clusters are not sufficient to guarantee binding by BEAF, and BEAF can bind sequences with a single CGATA [22]. In addition, less than one-third of dual-cores overlap with BEAF peaks. Thus details of what constitutes a high affinity BEAF binding site remain elusive.

A particularly interesting result to emerge from the BEAF mapping studies cited above is that over 85% of centers of BEAF peaks are within 500 bp of gene transcription start sites (TSSs), often within 200 bp. In turn, we found that around 85% of these BEAF-associated genes are found in lists of housekeeping genes [26, 27]. Thus, in addition to insulator activity, BEAF could play a role in keeping associated promoters active. Around half of BEAF peaks are between divergently transcribed genes. An example is scs’, which has two BEAF binding sites, one near each TSS. Around 20% of the dual-cores described above are also between divergently transcribed genes. At least 80% of these correspond to BEAF peaks, suggesting that BEAF might frequently bind near both TSSs of divergent genes. In contrast, less than 5% of dual-cores not near TSSs correspond to BEAF peaks. This is another illustration that clusters of CGATA motifs alone are not a good predictor of BEAF binding. However, there is no other known predictor and sequences with CGATA clusters near TSSs are often bound by BEAF.

To explore the role of BEAF in genome organization and gene regulation, we mapped long-distance interactions of four BEAF-binding regions using 4C. We chose high confidence BEAF binding sites that correspond to dual-cores located between divergently transcribed genes. The results were complicated, with one viewpoint in particular differing from the other three in terms of histone modifications, chromatin states and insulator proteins associated with interacting DNA. The strongest correlation was with housekeeping genes. BEAF is not essential for the observed interactions to occur. We conclude that long-distance interactions of active chromatin such as our viewpoints are highly variable and enriched for active chromatin. This is consistent with the model that condensed, largely inactive chromatin is sticky and associates into TADs that are separated by inter-TAD regions of active chromatin [27–29]. It is possible that phase-separation helps drive condensed chromatin into TADs [30, 31]. Redundant factors that open chromatin and promote transcription, perhaps including BEAF, would facilitate exclusion from TADs. The less sticky, active inter-TAD chromatin that fills the spaces between TADS could allow variable packing of TADs relative to each other, resulting in the variable long-distance interactions between active chromatin that we observe.

## Materials and methods

### 4C library preparation

4C libraries were prepared using published methods with modifications (S1 Fig) [32–34]. Two biological replicates were performed for each viewpoint. *Drosophila* Kc cells (Drosophila Genomics Resource Center Kc167) were grown at 25°C in Shields and Sang M3 medium (Sigma) with 1x Pen-Strep (Gibco) and 5% fetal bovine serum (Gibco). Cells (500 ml at ~9x10^6^ cells/ml) were spun down (400g, 4°C, 10 minutes) two times and resuspended in 30 ml PBS both times. Protein-DNA complexes were cross-linked by adding 37% formaldehyde (final concentration 1%) to the cells and incubating at room temperature for 10 minutes. Formaldehyde was quenched by adding 1.25 M glycine (final concentration 125 mM). The cells were washed with 30 ml PBS, resuspended in 10 ml lysis buffer (10 mM Tris-HCl pH 8.0, 10 mM NaCl, 0.2% NP40), and incubated on ice for 15 minutes. The cells were dounced 15 times on ice with a B pestle, incubated on ice for another 15 minutes, then dounced 15 times again. The nuclei were pelleted at 2000g at 4°C for 5 minutes.

Depending on the viewpoint, 5x10^8^ nuclei were resuspended in 500 μl DpnII (scs’, *hts*, *snf*) or NlaIII (*RpS6*) restriction enzyme buffer and distributed into 50 μl aliquots in 10 tubes. Nuclei were lysed by adding 10% SDS (final concentration: 0.3%) and shaking at 37°C overnight. SDS was diluted to 0.1% with DpnII or NlaIII restriction enzyme buffer, and the SDS was quenched by adding 20% Triton X-100 (final concentration 1.1%) and shaking at 37°C overnight. The following day 22.5 units of DpnII or 15 units of NlaIII (New England Biolabs) was added to each of the appropriate 10 tubes and shaken at 37°C overnight. The same amount of restriction enzyme was added the next day and shaken again at 37°C overnight. Restriction digestion efficiency was determined by isolating DNA from 5% of the digested samples to determine the DNA concentration and perform PCR using primers spanning viewpoint restriction sites (S1 Table), as described [34], and found to be approximately 90% cut.

Restriction digestions were stopped by adding SDS to 1.6% and heating at 65°C for 30 minutes. Samples cut with the same restriction enzyme were pooled, 30 μg was diluted to 2 ng/μl with ligation buffer, and 20% Triton X-100 was added to a final concentration of 1.1%. Samples were incubated at 37°C for 4 hours, then 6000 units T4 DNA ligase (New England Biolabs) was added and incubated at 4°C for 72 hours. After ligation, Proteinase-K was added to 0.1 mg/ml and incubated overnight at 65°C. Next samples were treated with 0.22 ng/ml RNAse A at 37°C for one hour, and DNA was extracted using phenol chloroform and ethanol precipitated. The DNA pellet was resuspended in 100 μl TE buffer and quantified by Qubit (Thermo-Fisher). We then set up second restriction digestions, using 3 μg per viewpoint. For DNA first cut with DpnII, we used CviQI (scs’) or NlaIII (*snf* and *hts*); for DNA first cut with NlaIII we used DpnII (*RpS6*). After the second digestion samples were phenol chloroform extracted, ethanol precipitated, resuspended, and 1 μg was ligated at 0.8 ng/μl at 16°C overnight. The final ligated DNA was again extracted, precipitated and quantified by Qubit. This DNA was the input for making 4C libraries.

The four 4C libraries were made by inverse PCR using Phusion polymerase (New England Biolabs) and primers with Ion Torrent PGM barcodes and adapters incorporated into the primer sequences (S1 Table). Two libraries were made for each of the four viewpoints, one for each end of a viewpoint. PCR products were size selected on a 2% agarose gel (100–500 bp). DNA from the gel was extracted with a Wizard PCR clean-up kit (Promega) and submitted to the LSU Genomics Facility for Ion Torrent sequencing. Data are available at NCBI GEO accession number GSE118013.

### Calculating significant *cis-* and *trans-*interactions using fourSig

Viewpoint sequences at the 5’ and 3’ ends were trimmed using Btrim [35]. The remaining sequences were aligned to the *Drosophila* Release 6.01 genome using bowtie2 [35, 36]. The resulting sam files were converted to bam files, filtering out sequences with Q-scores < 30, for use in the fourSig analysis suite [37]. Reads from both ends of viewpoints were combined for 4C analysis. In fourSig, bamToReTab.pl converted bam files to tab files, which are tables of reads mapped to restriction fragments generated by the first 4C restriction enzyme. The tab files were used by fourSig.R to calculate significant *cis-* and *trans-*interaction intervals. Additional information required for the script to run are: chromosomal location of the viewpoint, sliding window size, and FDR cutoff value. Significance thresholds for calculating FDR values were generated using 1000 random shuffling steps. We excluded ±2 kb from the viewpoint while calculating significant interactions. Sliding window sizes of 5 and 20 were used for *cis-* and *trans-*interactions, respectively, with an FDR value of 0.001 and an FDR probability value of 0.01. We used only Category 1 (broad) and Category 2 (intermediate) peaks in our analysis. Category 3 (narrow) peaks were excluded because they represent viewpoint interactions with a single restriction fragment, rather than also neighboring fragments. Therefore, they are more likely to be false positives. Overlap of *cis-* and *trans-*interactions from two biological replicates were calculated using a custom Python script.

### Performing virtual 4C using Hi-C data

To evaluate the quality of our 4C data, we performed virtual 4C on Hi-C data from DpnII-cut DNA. S2 cell data from GSE99104 [38] and GSE101317 [39], and Kc cell data from GSE85503 [28] and GSE80701 [40], were analyzed using HOMER (Hypergeometric Optimization of Motif EnRichment) software [41]. The analyzeHiC script was run with the -4C option using the coordinates of our 4C viewpoints with 2000 bp resolution and binning windows. Virtual 4C replicates were analyzed for reproducibility using BEDTools intersect (S2 Table) [42].

### Calculating overlap of significant *cis-* and *trans-*interactions with genomic features using GAT

Data for the genomic features used in Genomic Association Tester (GAT) analyses [43] were from published sources. Unless otherwise noted, all data are for *Drosophila* Kc cells. For data not available from Kc cells, we used data from S2 cells or embryos. These are reasonable substitutes since Kc and S2 cells are similar and are derived from embryos, and M1BP, Zw5 and TADs are more or less constitutive. The ChIP-seq files for H3K4me1, H3K4me3 and H3K27ac were obtained from GSE36374 [40]. BEAF, dCTCF, Su(Hw) and CP190 peaks were obtained from GSE15661 [24]. H3K27me3 depleted D and enriched E domains were obtained from GSE85504 [28], and the five chromatin states interval file was from GSE22069 [44]. We used housekeeping gene lists from the Akhtar and Razin labs [26, 27] and obtained S2 cell M1BP binding sites from GSE46630 [45]. Chromator (Chro), Zw5 (S2 cells), GAGA factor (GAF), H2Av, H3K9me2, H3K9me3 and H3K27me3 peaks were downloaded from modENCODE (datasets 277, 3803, 2568, 3282, 938, 3013 and 5136) [46]. Embryo TAD sites were from GSE34453 [47]. Genomic files in *Drosophila* genome Release 5 were converted to *Drosophila* genome Release 6 using the FlyBase Coordinate Converter [48]. For motif analysis, we used the FIMO program from the MEME suite to find motif occurrences in our viewpoint interactions [49] with dm6 release 6.01 as the reference sequence. We used consensus motifs curated from the literature by Mourad and Cuvier [50] and a p-value of 0.001. From the GAT output, we used fold enrichment (observed/expected nucleotide overlap) and Benjamini-Hochberg corrected *p*-values to generate heatmaps showing enrichment or depletion of features in 4C interaction partners of our viewpoints.

### Testing 4C interactions by 3C

To make 3C libraries, the 4C library protocol through the first ligation and purification of ligated DNA was followed. The 5’ primers were designed from the viewpoint sequence and the 3’ primers were designed from the interacting partner sequences (S3 Table). PCR was done using Phusion polymerase. PCR products were run on 1.5% agarose gels with a 50 bp DNA ladder, and the presence of a correct-sized PCR product indicated a positive 3C result. The interaction between scs’ and scs was used as a positive control [51].

### Testing 4C interactions by FISH

We used Fluorescence In Situ Hybridization (FISH) to test our 4C interactions [52]. A list of primers used to make FISH probes are in S4 Table. For each probe, we designed 4 or 5 primer pairs that give PCR fragments of 1.6–1.8 kb over 8–10 kb of the region of interest (viewpoint and partner). The PCR products were purified using a Wizard PCR clean-up kit (Promega). The PCR products for each probe were pooled in equimolar amounts and labelled with either Alexa Fluor 555 or Alexa Fluor 488 (Invitrogen FISH tag DNA kit). We used 90 ng of each labelled probe in 30 μl of FISH hybridization buffer per hybridization. For each hybridization, 10 wild-type *BEAF* (*y*^*1*^ *w*^*67c23*^) and 10 null *BEAF*^*AB-KO*^ [19] third instar larvae were dissected and processed as described [52]. BEAF-32B is essential, so the *BEAF*^*AB-KO*^ larvae were from heterozygous mothers that provided maternal BEAF to eggs. The FISH slides were viewed on a Leica TCS SP8 confocal microscope with a white light laser using a 63X water immersion lens, capturing Alexa Fluor 555, Alexa Fluor 488 and DAPI (DNA) images. Wing discs and brains were viewed.

### FISH image analysis using Fiji

FISH Z-stack images were analyzed using ImageJ Fiji [53]. Z-stacks were split into green (viewpoint), red (interacting partner) and blue (DNA) channels. The red and green channels were merged. Background noise was reduced by processing with the filter *Gaussian Blur* (value: 2.00) and removing noise using *Despeckle*. We then used the plugin ComDet, which marks colocalized red and green signals (yellow) with a yellow square [54]. A signal was considered colocalized if the red and green signals overlapped by ≥ 0.5 pixels. These marked images were again split into red, green and blue channels. The blue channel images were discarded, while images in the red and green channels were merged with the blue channel images from the original Z-stack images (before removing background noise). The merged images allow FISH signals to be assigned to specific nuclei. Finally, the percentage of colocalization of a viewpoint and its interacting partner was calculated after counting from 130 to 525 nuclei in wing discs and brains, in the presence and absence of BEAF.

## Results

### Selection of 4C viewpoints and initial characterization of interactions

**Fig 1:**
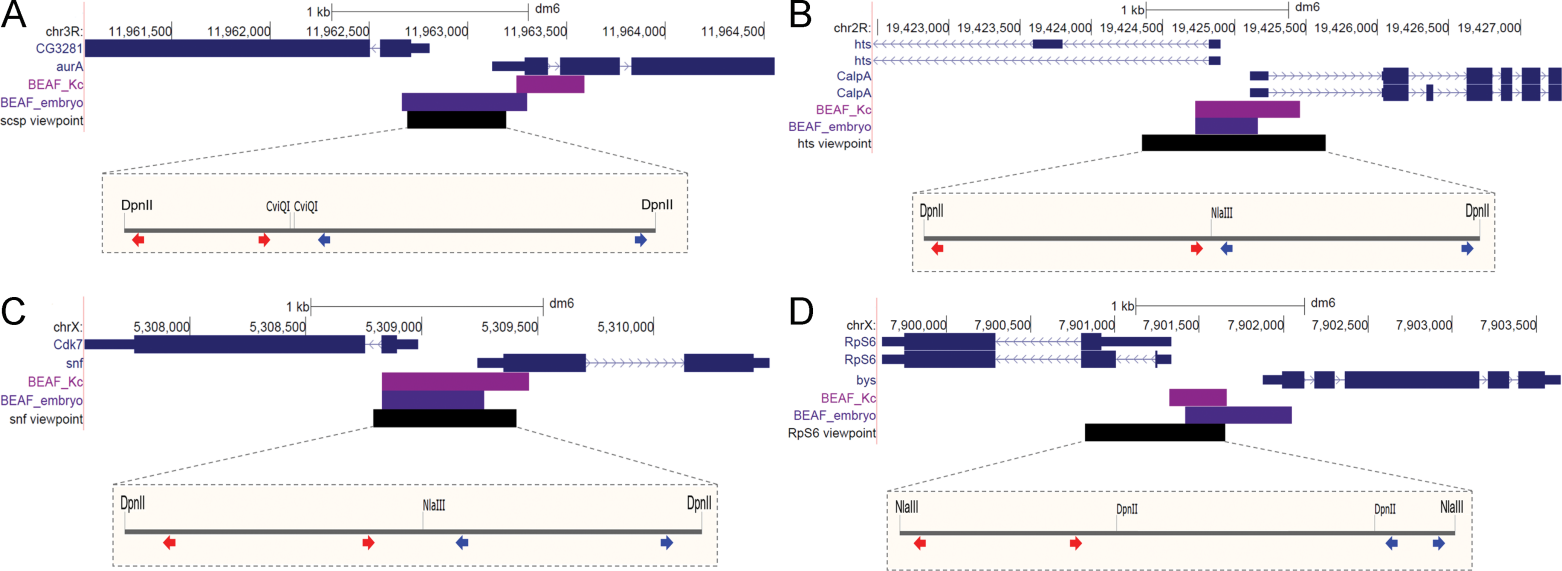
Locations of the 4C viewpoints. (**A**) Chr3R scs’ viewpoint at the divergent *CG3281* and *aurA* genes. (**B**) Chr2R *hts* viewpoint at the divergent *hts* and *CalpA* genes. (**C**) ChrX *snf* viewpoint at the divergent *Cdk7* and *snf* genes. (**D**) ChrX *RpS6* viewpoint at the divergent *RpS6* and *bys* genes. The UCSC Genome Browser snapshots show the gene models (blue), BEAF peak limits from Kc cell (magenta) or embryo (purple) mapping, and the viewpoint region (black). The expanded views show the sites for the two restriction enzymes used, with the positions of inverse-PCR primers for the left and right sides of the viewpoints indicated by red and blue arrow pairs respectively.

Insulator binding proteins are also called architectural proteins because they are thought to play a role in 3D genome architecture by mediating long-distance chromatin looping interactions [55]. We wanted to determine the nature of long-distance interactions made by regions that BEAF binds to, and whether BEAF is necessary for these looping interactions to occur. For this purpose we chose 4 BEAF binding regions from our previous embryo mapping results to use as 4C viewpoints [22]. Approximately 85% of the 1818 regions we mapped were within 300 bp of TSSs, and half were between divergently transcribed genes. The 4 regions we chose are between divergently transcribed genes (Fig 1, S2 Fig) and have CGATA motifs that fit the dual-core model [21]. In addition, BEAF binds to these regions in datasets generated by other labs, including another embryo dataset, Kc cells, Mbn2 cells, and S2 cells [23–25]. Kc cell data from modENCODE indicates that Chromator and CP190 are also found at these viewpoints, and ZIPIC is found at the hts and RpS6 viewpoints [56].

**Fig 2:**
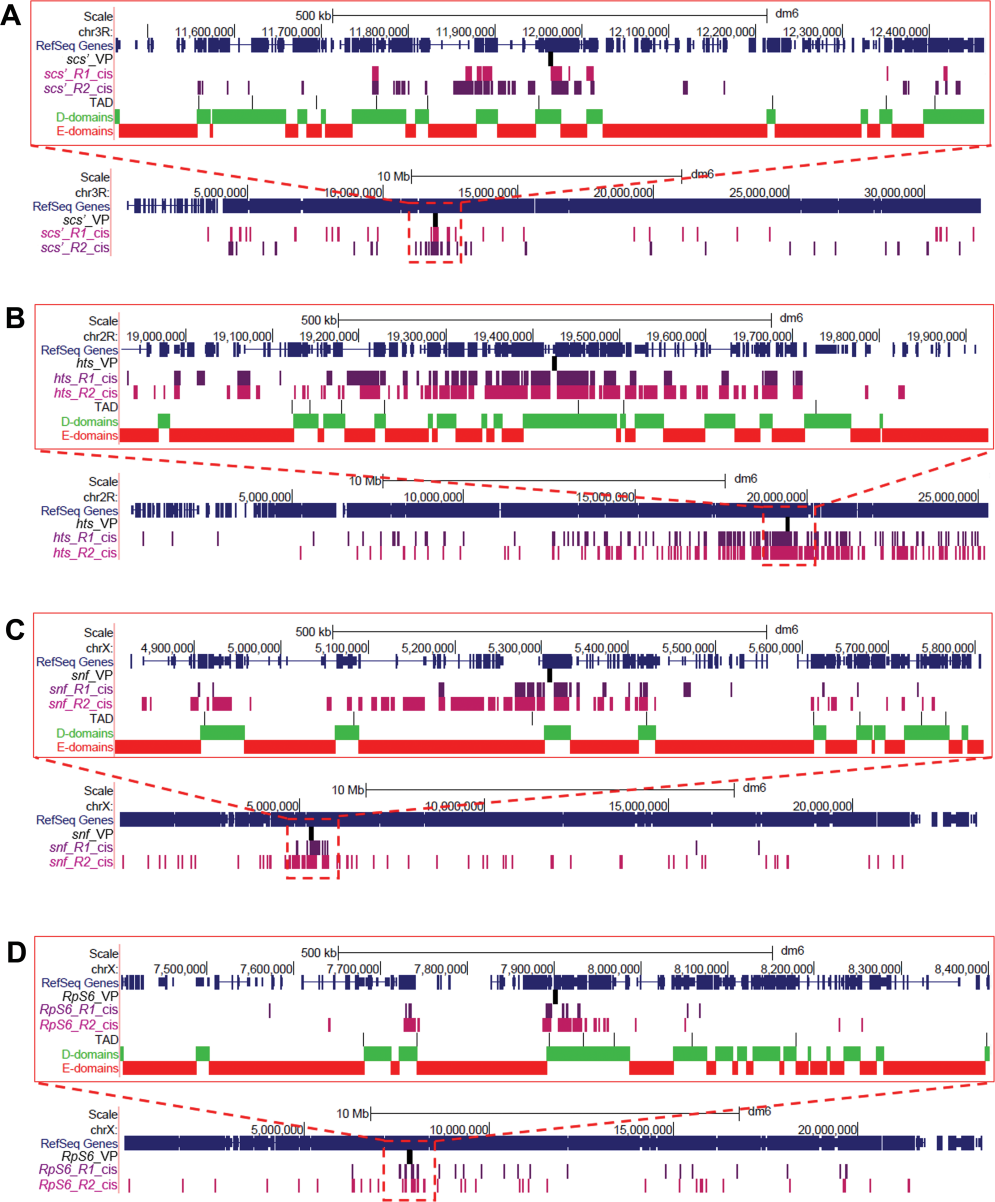
Distribution of *cis*-interactions along the chromosome arms of the viewpoints. Shown are UCSC genome browser snapshots of near-*cis*-interactions (1 Mb centered on the viewpoints, top of each panel) and all interactions along the chromosome arm containing the viewpoint (bottom of each panel) for replicates R1 (purple) and R2 (magenta) for the (**A**) scs’, (**B**) *hts*, (**C**) *snf*, and (**D**) *RpS6* viewpoints, with the viewpoint location indicated (VP). Near-cis-interactions also show the gene distribution (blue), embryo TAD boundaries, and H3K27me3-depleted D domains (green) and enriched E domains (red). Most interactions common between replicates are in the near-*cis* regions, which also contain many unique interactions.

**Fig 3:**
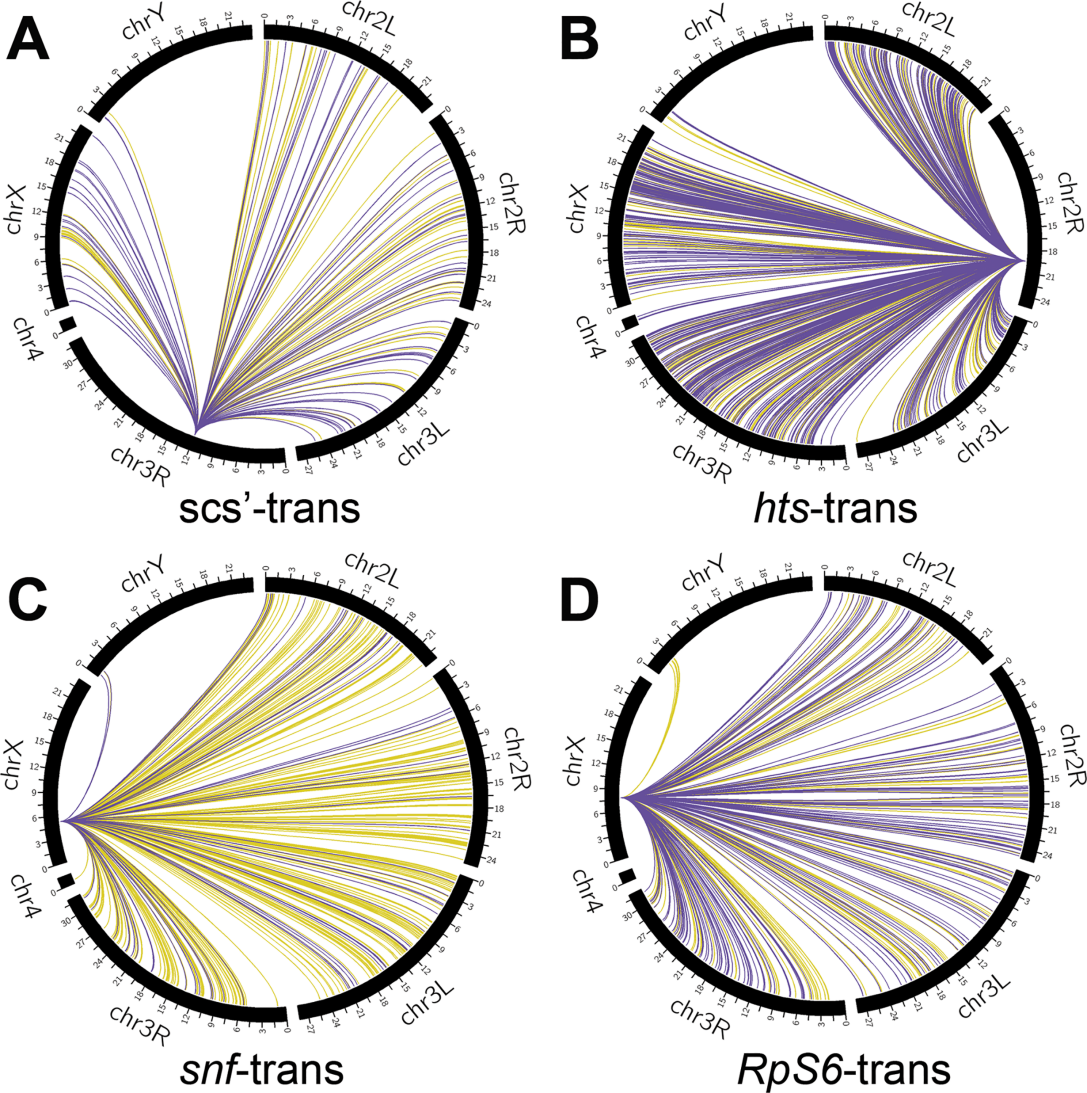
Circos-plots of significant *trans-*interactions. Interactions of replicates R1 (yellow) and R2 (purple) occurring on chromosome arms other than the one containing the viewpoint are shown for the (**A**) scs’, (**B**) *hts*, (**C**) *snf*, and (**D**) *RpS6* viewpoints. There were 295 *trans*-interactions that overlapped for the *hts* replicates, while there was only 1 overlapping *trans*-interaction for the replicates of the scs’, *snf* and *RpS6* viewpoints.

We obtained 1.4 million to 32 million filtered, aligned reads per viewpoint per replicate (median 2.5 million), of which around 30% mapped to the viewpoint chromosome arm (S5 Table). This level of inter-chromosomal ligation is reasonable for open chromatin, such as our viewpoints, when using a 4-cutter restriction enzyme [57]. The number of reads mapping to significant 4C interactions by fourSig varied between replicates and viewpoints from 13% to 56% for both *cis* and *trans*-interactions. Hundreds of *cis*-interactions were identified (Fig 2A–2D). In line with other 4C results, there were high densities of interactions near the viewpoints (S3 Fig). Most interactions that overlapped between replicates were found in a window of 1 Mb centered on the viewpoints, which we refer to as near-*cis*-interactions (Table 1). Low levels of overlap of *cis*-interactions between replicates outside of this window, which we refer to as far-*cis*-interactions, were only observed for the *hts* and *RpS6* viewpoints. Although the *snf* and *Rps6* viewpoints are both on the X chromosome, none of their interactions overlapped with each other even though they are only separated by around 2.5 Mb and both had longer-distance *cis*-interactions. Similarly, hundreds of *trans*-interactions were identified (Fig 3A–3D, Table 1). There was around 15% overlap for replicates of the *hts* viewpoint, but less than 1% overlap of *trans*-interactions for replicates of the other viewpoints. Additionally, only a few *trans*-interactions for any viewpoint overlapped with interactions for other viewpoints.

**Table 1:** 4C interaction data.

Viewpoint	Number of *cis*-interactions	Common *cis*-interactions (%)	Common interactions that are near*-cis (*%*)*	Unique interactions that are near-*cis* (%)	Number of *trans*-interactions
scs'_R1	270	22	100	59	386
scs'_R2	148	33	100	29	257
hts_R1	876	58	74	15	2110
hts_R2	1216	42	76	30	1418
snf_R1	140	79	100	80	190
snf_R2	423	27	100	53	951
RpS6_R1	76	45	94	17	517
RpS6_R2	206	21	95	53	374

*Cis*-interactions occur on the same chromosome arm as the viewpoint; *trans*-interactions are on any other chromosome arm. Common interactions are interactions that overlap in both biological replicates (R1 and R2) for a viewpoint. Near-*cis*-interactions are interactions that occur within 500 kb on either side of a viewpoint.

### Virtual 4C and 3C analysis of 4C interactions

There was little overlap between viewpoint replicates outside of the near-*cis* region. The low level of reproducibility could reflect the complexity of long-distance interactions that occur if chromosome packaging is highly variable between nuclei. Alternatively, it is possible that our viewpoints make a limited number of preferred long-distance *cis*- and *trans*-interactions either due to affinity for particular regions or limited variability in nuclear organization. In the latter case, the variability in 4C interactions that we detected would indicate problems with our 4C libraries. We took two approaches to explore this. In one, we performed virtual 4C using Hi-C data. In the other, we performed 3C analysis of selected interactions.

We performed virtual 4C on four Hi-C datasets. All were from DpnII-cut DNA, with two from Kc cells and two from S2 cells. One Kc cell dataset only gave near-*cis* interactions, and was not analyzed further [40]. The other datasets [28, 38, 39] were similar to our 4C data in that many viewpoint interactions, especially interactions further than 500 kb away, were unique to one replicate (S2 Table). This indicates that the lack of reproducibility of distant interactions in our 4C data is a common feature of these types of experiment.

Interactions found in the 4C analyses were confirmed by 3C for all viewpoints (Table 2, S3 Table). This included the previously reported interaction between the scs’ and scs insulators [51]. Between 13 and 16 *cis*-interactions were tested for each viewpoint, and over half gave a positive 3C result for each. Over 80% of the tested interactions closer than 1 Mb were positive by 3C (24 of 29), while over 50% of the tested interactions ranging from 1 Mb to 17.1 Mb from viewpoints were positive (15 of 28). Most of the negative 3C results (72%) were for interactions over 1 Mb distant from viewpoints (S3 Table). In addition, 3 viewpoints had 3 *trans*-interactions tested, and 56% were positive by 3C. Similar to finding that many 4C interactions were detected in only one biological replicate, around 57% of the 3C interactions were detected in only one 3C replicate. Presumably this reflects the variability of nuclear organization, so no 3C or 4C library captures all interactions made by a viewpoint. In contrast, no cross-interactions were detected between viewpoints and interaction partners from a different viewpoint. Seven or eight partners were cross-tested for each viewpoint, including both *cis* and *trans*-interactions (Table 2, S3 Table). Together, this suggests that most 4C interactions called by fourSig are valid.

**Table 2:** Results of testing 4C interactions by 3C.

Viewpoint	*cis* < 1 Mb		*cis* > 1 Mb		*trans*		Cross-check	
	No.	% 3C pos.	No.	% 3C pos.	No.	% 3C pos.	No.	% 3C pos.
scs'	9	67	4	75	0	na	8	0
hts	5	100	10	30	3	67	8	0
snf	7	86	6	50	3	33	7	0
RpS6	8	88	8	75	3	67	7	0

The number (No.) of tested *cis*-interactions within 1 Mb on either side of the viewpoints (*cis* < 1 Mb), further than 1 Mb (*cis* > 1 Mb), and *trans*-interactions are indicated together with the percent that gave a positive 3C result (% 3C pos.). Cross-check: testing a viewpoint with an interaction from a different viewpoint; na: not applicable. See S3 Table for details.

### FISH analysis of 4C interactions

**Fig 4:**
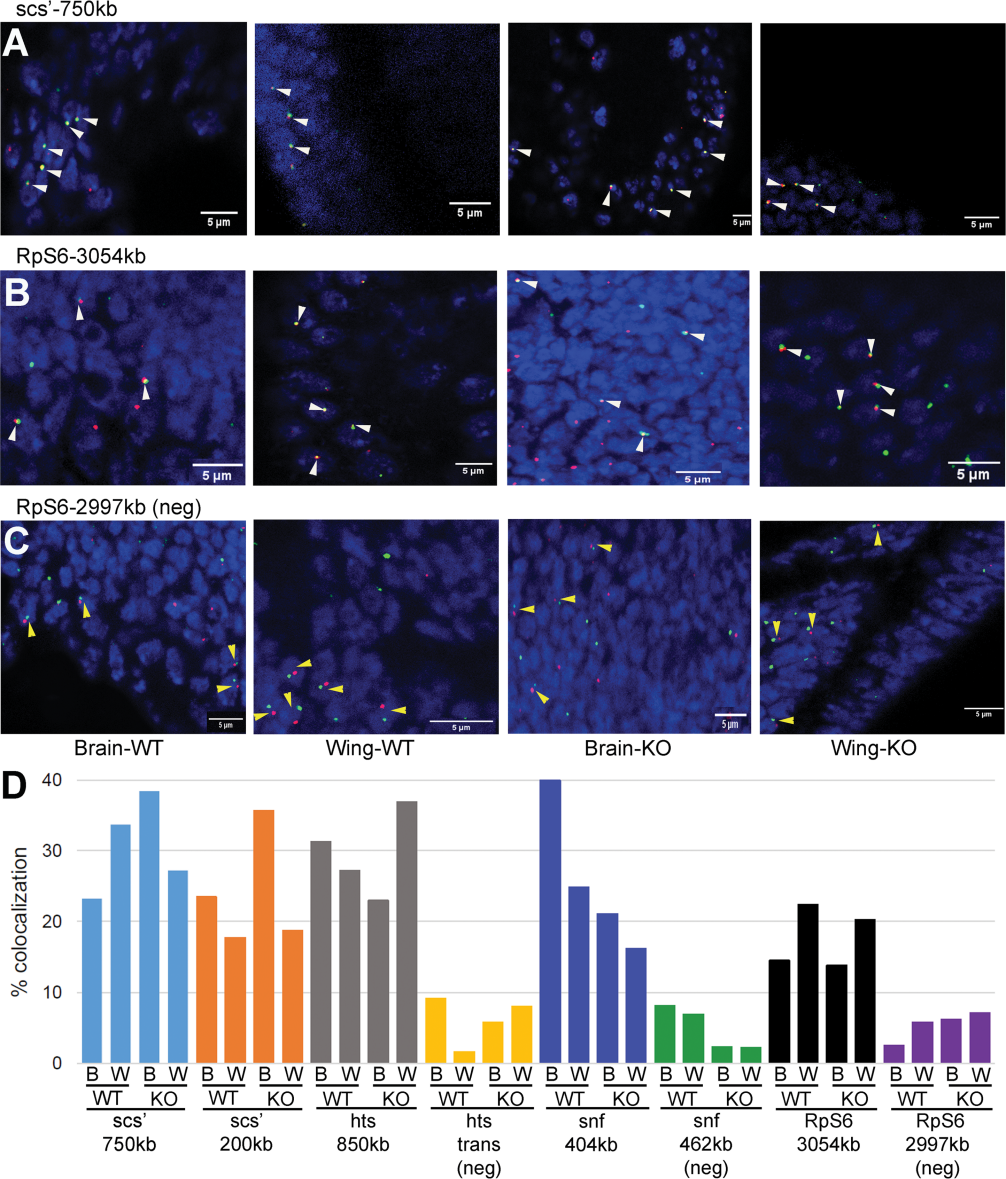
Testing 4C interactions by FISH. Representative confocal micrographs of hybridizations to third instar larval brains and wing discs from *BEAF* wild-type (WT) or null (KO; *BEAF*^*AB-KO*^) animals are shown, as labeled. DNA is labeled with DAPI to identify nuclei. White and yellow arrowheads indicate representative nuclei with colocalizing or non-colocalizing FISH signals, respectively. (**A**) Hybridization to the scs’ viewpoint (green) and a sequence around 750 kb away (red). Overlapping signals are yellow. (**B**) Hybridization to the *RpS6* viewpoint (green) and a sequence around 3054 kb away (red). (**C**) Negative control hybridization to the *RpS6* viewpoint (green) and a sequence around 2997 kb away (red) that does not represent a 4C interaction. (**D**) Graph of all FISH results, indicating % of counted nuclei showing colocalization of signals (n = 131 to 525, see S6 Table). The viewpoint probe and distance to the second probe are indicated, as well as if the second probe was a negative control (neg) that did not represent a 4C interaction. W: third instar larval wing disc; B: third instar larval brain; WT: wild-type *BEAF* animal; KO: null *BEAF*^*AB-KO*^ animal. The percent colocalization is comparable between WT and KO for both wing discs and brains, but is lower for the three negative controls.

Fluorescence in situ hybridization (FISH) was also used to examine interactions found by 4C. Interactions verified by 3C were selected for analysis by FISH. Two interactions were tested for the scs’ viewpoint, and one for each of the others. Interactions ranged from 200 kb to 3 Mb away from viewpoints. Three negative controls were included. One used a chromosome 3R partner of scs’ with the *hts* viewpoint, representing a potential *trans*-interaction. The other two tested for *cis*-interactions on the X chromosome, switching the *snf* and *RpS6* viewpoints and partner sequences. Hybridization was done with third instar larval wing discs and brains. To determine if BEAF is required for the detected 4C interactions to occur, we used wild-type larvae and larvae homozygous for the null *BEAF*^*AB-KO*^ allele [19]. Null females are nearly sterile, so the null larvae were from heterozygous mothers. BEAF is maternally provided, but is depleted to undetectable levels in null third instar larvae [19]. Between 130 and 525 nuclei were counted for each of the conditions (S6 Table). The expected *cis*-interactions were confirmed by FISH for all four viewpoints, while the negative controls gave obviously lower colocalization of FISH signals (Fig 4). However, the results indicate that BEAF is not required for the interactions to occur. The lack of dependence on BEAF is consistent with a recent report that RNAi knockdown of BEAF has minimal effects on TAD organization detected by Hi-C [58].

### Feature analysis of 4C interactions for epigenetic marks and DNA binding motifs

**Fig 5:**
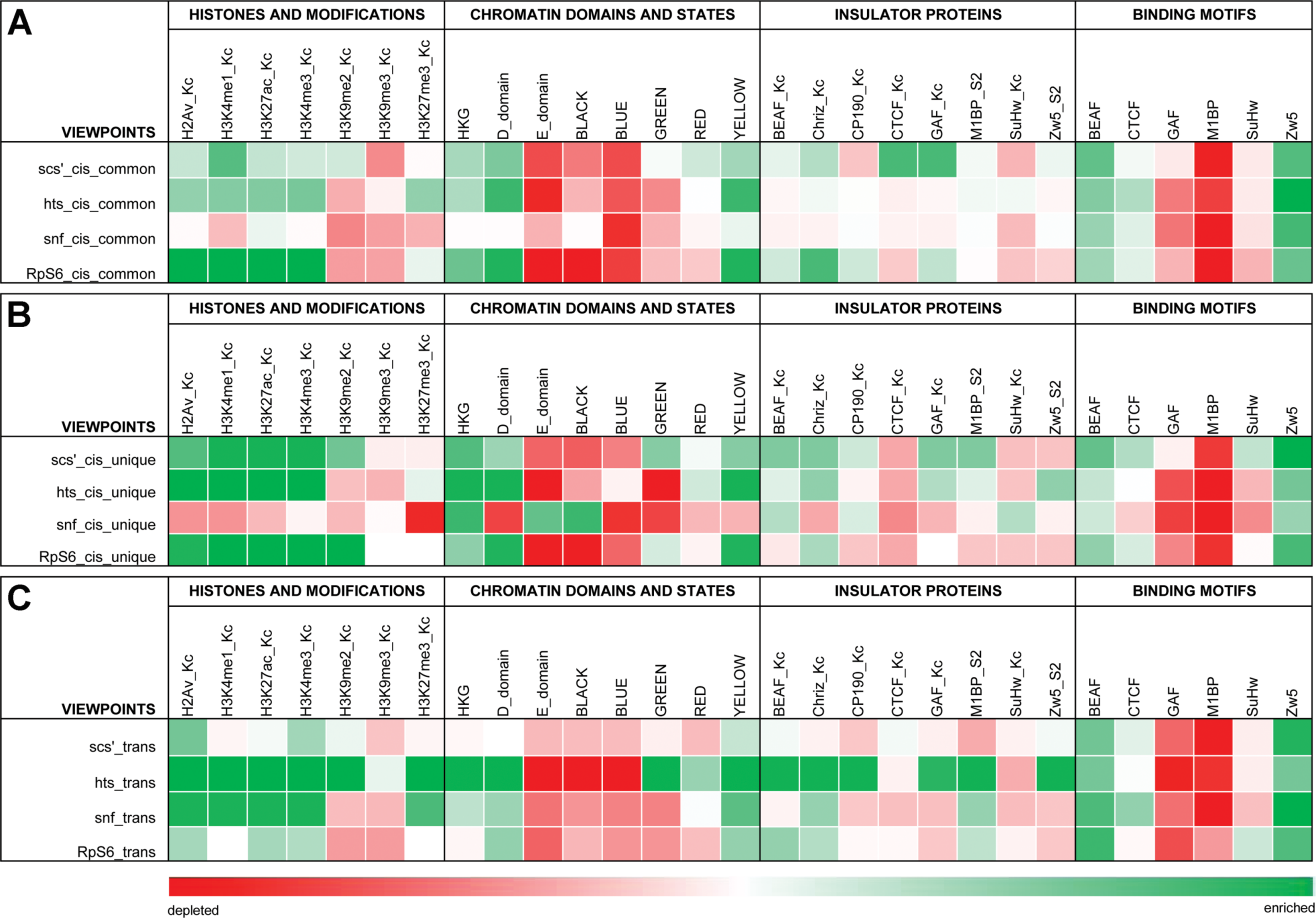
Feature analysis shows the 4C interactions have a preference for active chromatin and housekeeping genes. The 4C interactions were categorized as (**A**) overlapping between 4C replicates (*cis*-common) or (**B**) unique *cis*-interactions or (**C**) *trans*-interactions (nearly all unique) for each viewpoint. Genomic Association Tester (GAT) analysis was done for the indicated histones and modifications, chromatin states and domains, housekeeping genes, and insulator proteins plus M1BP. The FIMO program from the MEME suite was used to find DNA binding motif occurrences in viewpoint interactions. Enrichment is indicated in green, depletion in red. See text for details.

We wanted to know if interactions with our viewpoints were enriched for particular genomic features. BEAF is mostly present near TSSs and most associated genes are actively transcribed [22]. We chose our viewpoints based on this. Therefore we postulated the interaction partners of our viewpoints would also be enriched for active histone marks, especially the active promoter mark H3K4me3. We focused on this mark, H3K4me1 (enhancers), H3K27ac (active enhancers), H2Av (active chromatin), and for inactive chromatin we used the repressive histone marks H3K9me2, H3K9me3 and H3K27me3. We split interactions into three categories. *Cis*-common refers to *cis*-interactions present in both 4C replicates (mostly near-*cis* as described above, Fig 2); *cis*-unique refers to *cis*-interactions present in only one replicate; and *trans*-interactions, nearly all of which were unique. As shown in Fig 5, we found that all interactions for the scs’, *hts* and *RpS6* viewpoints are generally enriched for active histone marks and depleted for inactive histone marks. On the other hand, the *snf* viewpoint follows this pattern for *trans*-interactions but *cis*-interactions are generally depleted of active as well as inactive marks used in our analysis. It should be noted that *cis*-common and *cis*-unique interactions had similar associations for a given viewpoint. Like our virtual 4C and 3C results, this similarity suggests that unique interactions, many of which are far from viewpoints, represent genuine interactions.

Interactions were also characterized with respect to a five chromatin state model based on various histone marks and chromatin proteins [44]. Interactions for all viewpoints were enriched for the active yellow chromatin state, and sometimes to a lesser extent also the red state (Fig 5). Yellow chromatin is enriched for broadly expressed (housekeeping) genes, while the red state is enriched for genes that show more restricted expression patterns (developmental). Conversely, interactions were mainly depleted for repressed black (devoid of histone modifications) and Polycomb group (PcG)-associated blue (H3K27me3) states. *Cis*-interactions for the *snf* viewpoint are an exception to this, particularly the *cis*-unique interactions. They are enriched for black chromatin and depleted for yellow and red, consistent with the depletion for histone modifications. The repressive HP1-associated green chromatin state (H3K9me2/3) is more complicated. *Trans*-interactions for the *hts* viewpoint are enriched for this state, as well as for yellow and red, while *cis*-unique interactions for the scs’ viewpoint and to a lesser extent the *RpS6* viewpoint also show some enrichment for green chromatin. A possible explanation for this rather unexpected association could be that active genes in repressive green chromatin are kept active in part by long-distance interactions with active genes [59].

As another way to view the chromatin in our 4C interactions, we analyzed it with respect to D and E domains [28] and housekeeping genes [26, 27]. D domains are depleted for the PcG-associated mark H3K27me3, are largely transcriptionally active, and are enriched for housekeeping genes. In contrast, E domains are enriched for H3K27me3, are largely transcriptionally inactive, and are enriched for regulated genes. Consistent with usually being found near TSSs of housekeeping genes, we found that 95% of BEAF peaks in embryos and Kc cells are in D domains [22, 24]. Our 4C interactions are also associated with D domains and housekeeping genes, and depleted for E domains (Figs 2 and 5). These associations are weakest for scs’ *trans*-interactions. Also, once again *snf cis*-interactions varied by having a stronger association with E domains and weaker association with D domains than other viewpoints, although it is similarly associated with housekeeping genes.

Finally, we checked to see if our 4C interactions were enriched for insulator proteins or their DNA binding motifs (Fig 5). We used publicly available Kc cell datasets for BEAF, dCTCF, Su(Hw), CP190, Chro and GAF, and an S2 cell dataset for Zw5. In addition, we used an S2 cell dataset for M1BP because, like BEAF, it often localizes near TSSs of housekeeping genes [45]. As explained in Methods, Kc datasets were not available for Zw5 or M1BP. We found a 28% overlap of Kc cell BEAF peaks with M1BP peaks (45% overlap of M1BP with BEAF), suggesting that BEAF and M1BP independently bind near housekeeping gene TSSs.

Interactions were enriched for M1BP and insulator proteins other than Su(Hw), although there is no clear pattern. In general, the most consistent associations were with BEAF and Chro, followed by M1BP, GAF and finally CP190. However, for each viewpoint there were differences in association profiles between *cis*-common, *cis*-unique and *trans*-interactions. Once again, association profiles for interaction partners of the *snf* viewpoint differed the most from those of the other viewpoints. Analysis for DNA binding motifs was more consistent, with all viewpoints showing a general enrichment for BEAF and Zw5 motifs, a depletion for GAF, M1BP and Su(Hw) motifs, and being fairly neutral for dCTCF motifs. The significance of the difference between the ChIP and motif analyses is unclear.

We conclude that rather than any particular protein or histone modification, interactions with our viewpoints are driven by redundant factors that lead to open chromatin conducive to active transcription, particularly of housekeeping genes. On the surface, it appears the *snf* viewpoint often interacts with different chromatin environments than the other viewpoints. Yet *snf* viewpoint interactions are enriched for housekeeping genes. This suggests that active transcription is a unifying theme even if surrounded by a generally repressive environment.

## Discussion

Early Hi-C mapping of chromosome interactions found that chromatin is organized into TADs [47, 60, 61]. There is strong evidence that the insulator protein CTCF, together with Cohesin, plays a role in defining boundaries between TADs in vertebrates by binding oriented binding sites [1, 3, 62, 63]. *Drosophila* has several insulator proteins in addition to a homolog of vertebrate CTCF, and it has been proposed that insulator proteins often cluster at *Drosophila* TAD boundaries with boundary strength correlating with the number of clustered proteins [64]. Other evidence suggests that rather than TAD boundaries, *Drosophila* has inter-TADs that are regions of clustered housekeeping genes [27, 40]. This is reflected in polytene chromosome organization [29]. The short-range interactions within these inter-TADs presumably limits interactions between adjacent TADs. It is possible that housekeeping genes also form some TAD boundaries or inter-TADs in vertebrates [60, 65].

BEAF was originally identified as an insulator binding protein. Genome-wide mapping revealed that it usually binds near TSSs, mainly of housekeeping genes. Our intention was to determine how BEAF contributes to nuclear architecture by mapping looping interactions made by BEAF-associated sequences. Rather than focus only on near-*cis*-interactions, we were interested to characterize longer distance interactions to determine if there were any shared genomic features that might drive these interactions. Our FISH results indicate that BEAF is not essential for the interactions that we detect, consistent with recently observed minimal effects of BEAF RNAi knockdown on TAD organization [58]. There could be residual maternal BEAF present in third instar larvae, although our efforts to detect it were not successful. There is certainly some BEAF left in cells after RNAi, although in the same study a similar knockdown of M1BP had drastic effects on chromosome organization [58]. It is possible that residual BEAF could be sufficient to maintain chromatin organization, or that there is an epigenetic memory established by BEAF. For example, maternal BEAF could play a role in zygotic activation of associated housekeeping genes and afterwards not be necessary to keep the genes active. In our view, it is more likely that BEAF is one of several redundant factors working in various combinations to open and keep associated promoters active. In fact, rather than a direct role in long range nuclear architecture, the major role of BEAF might be to help open and keep promoter regions in an active state. Regardless of the role of BEAF, our results point to principles of long-distance interactions made by active chromatin.

Viewpoints were selected that were typical of many BEAF binding regions in that they had multiple 32B binding motifs between divergently transcribed genes. Based on high-throughput expression data, seven of the eight genes are classified as housekeeping [26, 27]. The exception is the *aurA* gene in the scs’ insulator; *aurA* is active in cultured cells. As such, the viewpoints are in active chromatin. Interactions made by three of the viewpoints are enriched for active chromatin marks, the active yellow chromatin state that is enriched for housekeeping genes [44], H3K27me3-depleted D domains that are enriched for housekeeping genes [28], and housekeeping genes. This indicates active chromatin preferentially interacts with active chromatin. *Trans*-interactions for the *snf* viewpoint follow this pattern, but *cis*-interactions are generally depleted of histone modifications and are generally enriched for the inactive black chromatin state and repressive H3K27me3-enriched E domains. Yet interactions are also enriched for housekeeping genes. One interpretation is that active chromatin in generally repressive environments is on the surface of inactive domains, available for long-distance interactions with other active chromatin such as our viewpoints. This could also explain the enrichment of interactions for other viewpoints with the HP1-associated repressive green chromatin state, in addition to their association with the active yellow chromatin state and D domains.

Although we found a tendency for interactions to occur with active chromatin enriched for housekeeping genes, we did not find evidence that a particular insulator protein drives the interactions. This suggests that redundant factors involved in promoting active transcription participate in establishing the long-distance interactions we found. These factors are likely to include BEAF, Chro, M1BP, CP190 and GAF. Of these, GAF is surprising because it is thought to be associated with regulated gene expression and pausing by RNA polymerase II, although a correlation with BEAF was previously noted [66].

**Fig 6:**
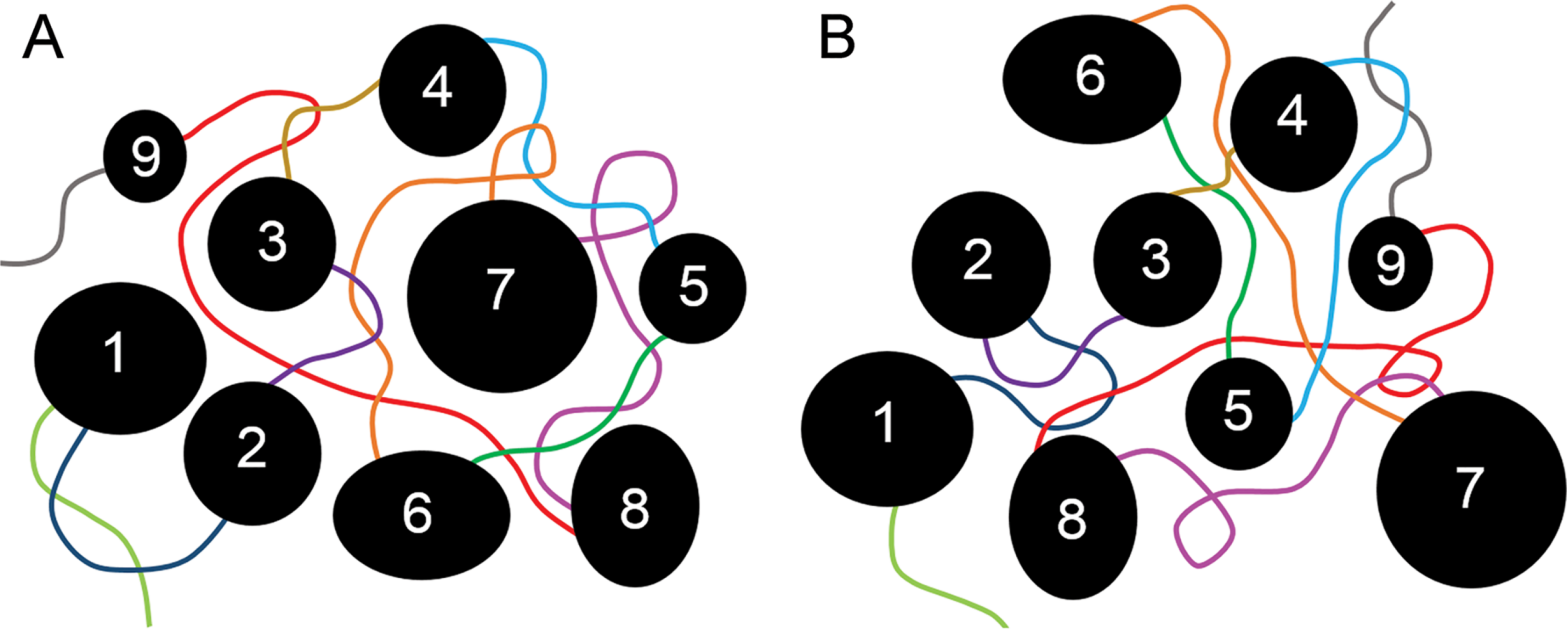
Model for variable long-distance interactions between active chromatin. In this model, TADs (numbered black circles and ovals) are regions of mainly condensed, inactive chromatin that are largely reproducible between cells and cell types. They are separated by inter-TAD regions (lines of different colors) of open, active chromatin such as housekeeping genes. While interactions within TADs are fairly reproducible, packing of TADs relative to each other is highly variable as indicated by the two examples in (**A**) and (**B**). Inter-TADs fill the space between TADs, and make variable long-distance interactions (crossing lines of different colors) depending on how TADs are arranged relative to each other. Active chromatin within TADs could be mainly on the surface of TADs (for simplicity, not shown) and so also participate in active chromatin interactions. Highly transient interactions could keep active chromatin mobile, increasing the variability of contacts. This model is particularly appealing if condensed chromatin has a tendency to phase-separate from active chromatin.

Few of the *cis*-interactions further than a few hundred kilobases from our viewpoints or *trans*-interactions were reproducible between replicates. Yet our 4C interactions represent multiple restriction fragments in the neighborhood ligating to our viewpoints, and 3C and FISH results suggest that most are likely genuine interactions. This is supported by virtual 4C analysis of our viewpoints using Hi-C data, which also found a tendency for long-distance interactions to be unique to one replicate. This indicates that long-distance interactions are highly variable, and individual 4C libraries do not capture all interactions. Why would TADs be fairly reproducible between cells, cell types and species while long-distance interactions of active chromatin are not? Repressive chromatin is thought to be sticky, and to be largely responsible for generating TADs [27, 28]. It is possible that these sticky interactions could cause phase separation of heterochromatin from decondensed, active chromatin [30, 31]. Packing TADS together could be less reproducible than folding within TADs, resulting in active chromatin, which is less sticky and between TADs, having different long-distance neighbors in different cells (Fig 6). Highly transient interactions in this environment could keep active chromatin mobile, increasing the number of contacts that can be captured by cross-linking.

Although our viewpoints share BEAF binding, divergent transcription and housekeeping genes, interactions of the *snf* viewpoint showed some differences from the other three. This raises the question of whether there are different subtypes of active chromatin interactions involving housekeeping genes. For instance, many of the original TADs defined in embryos have BEAF binding near their borders [47]. None of our viewpoints are included in that set. However, higher resolution analyses of *Drosophila* TADs have been done. One found over 2800 TADs [58]. Using their Chorogenome Navigator, we found that two of our viewpoints, *snf* and *hts*, correspond to TAD boundaries (S2 Fig). Looking at the Hi-C heatmaps, these regions could also be considered inter-TADs. Another found over 4000 TADs [39]. All of our viewpoints are near TAD boundaries in that analysis. Thus as resolution increases, more of our viewpoints are placed at TAD boundaries (or TADs and inter-TADs are divided into micro-TADs). This dependence on resolution could reflect some aspect of chromatin that is different between different regions where BEAF is found. At this point it is not clear why the *snf* viewpoint interacts with chromatin with some different characteristics than the other three viewpoints. It remains for future studies to determine what drives the complex folding of genomes beyond the level of TADs. Our results suggest that one factor could be the preference for active chromatin containing housekeeping genes to interact, perhaps driven in part by exclusion from sticky, condensed chromatin.

## Supporting information

S1 Fig Schematic of 4C library preparation. Drosophila Kc cells were cross-linked with formaldehyde, lysed, and digested using a restriction enzyme recognizing 4 bp. The protein-DNA complexes were diluted so that the sticky DNA ends were ligated in conditions that favor intramolecular ligations. Cross-links were reversed and DNA was isolated. Because multiple DNA fragments could be ligated into large circles, a second digestion was done with another restriction enzyme recognizing 4 bp. Ligation was again performed under dilute conditions to favor intramolecular ligations. Resulting circles were subjected to inverse PCR using primers based on DNA sequences in viewpoints of interest, to co-amplify unknown sequences from interaction partners. Our primers included Ion Torrent A and P1 adapters and barcode sequences. Amplified 4C libraries were size selected and used for high-throughput sequencing.
(PDF)

S2 Fig Chorogenome Navigator snapshots of 1 Mb regions around the viewpoints. Shown are the Hi-C maps, genome-wide mapping information for various insulator and other chromatin proteins (BEAF, M1BP, ZIPIC, dCTCF, Su(Hw), Ibf1/2, Pita, GAF, Zw5, condensin Cap-H2, CP190, Chromator, cohesion Rad21, Pol II) and histone modifications (H3K36me3, H3K79me3, H3K27me3, H4K16ac, H3K4me1, H3K4me3), binding site motif locations (BEAF, M1BP, motif-6, motif-8, ZIPIC, dCTCF, Su(Hw), Ibf) and gene models around the (**A**) scs’ viewpoint (*CG3281* and *aur* genes); (**B**) *hts* viewpoint (*hts* and *CalpA* genes); (**C**) *snf* viewpoint (*Ckd7* and *snf* genes); and (**D**) *RpS6* viewpoint (*RpS6* and *bys* genes). See Chorogenome Navigator (http://chorogenome.ie-freiburg.mpg.de/) [52] for details.
(PDF)

S3 Fig 4C read distributions in 1 Mb regions centered on viewpoint regions. The highest read densities are around the (**A**) scs’ viewpoint, (**B**) *hts* viewpoint, (**C**) *snf* viewpoint, and (**D**) *RpS6* viewpoint for both biological replicates, as is typical of 4C studies.
(PDF)

S1 Table Primers for checking restriction digestion efficiency while making 4C libraries, and inverse PRC primers for making 4C libraries. (PDF)

S2 Table Virtual 4C analysis of viewpoints using the indicated Hi-C data. (PDF)

S3 Table Primers for 3C validation of 4C interactions, and 3C results. (PDF)

S4 Table Primers for preparing FISH probes. (PDF)

S5 Table Sequence read and alignment information, and percent of reads in significant 4C *cis*- and *trans*-interactions. (PDF)

S6 Table FISH nuclei count data, corresponding to Fig 4. (PDF)
