# Supplementary material for: 4C-seq characterization of Drosophila BEAF binding regions provides evidence for highly variable long-distance interactions between active chromatin
Source: PLoS One. 2018 Sep 24;13(9):e0203843. doi: 10.1371/journal.pone.0203843 (PMC6152978; doi:10.1371/journal.pone.0203843)
Supplement: S1 Fig — Drosophila Kc cells were cross-linked with formaldehyde, lysed, and digested using a restriction enzyme recognizing 4 bp. The protein-DNA complexes were diluted so that the sticky DNA ends were ligated in conditions that favor intramolecular ligations. Cross-links were reversed and DNA was isolated. Because multiple DNA fragments could be ligated into large circles, a second digestion was done with another restriction enzyme recognizing 4 bp. Ligation was again performed under dilute conditions to favor intramolecular ligations. Resulting circles were subjected to inverse PCR using primers based on DNA sequences in viewpoints of interest, to co-amplify unknown sequences from interaction partners. Our primers included Ion Torrent A and P1 adapters and barcode sequences. Amplified 4C libraries were size selected and used for high-throughput sequencing. (PDF) [file pone.0203843.s001.pdf]

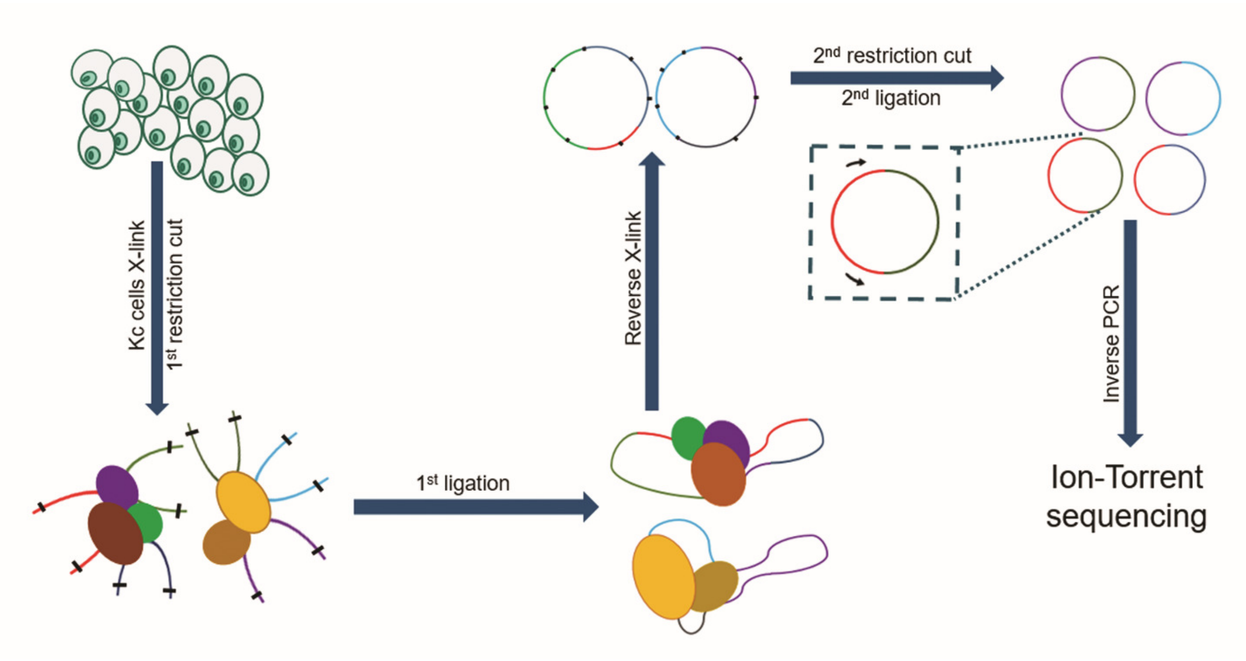

S1 Fig: Schematic of 4C library preparation. Drosophila Kc cells were cross-linked with formaldehyde, lysed, and digested using a restriction enzyme recognizing 4 bp. The protein-DNA complexes were diluted so that the sticky DNA ends were ligated in conditions that favor intramolecular ligations. Cross-links were reversed and DNA was isolated. Because multiple DNA fragments could be ligated into large circles, a second digestion was done with another restriction enzyme recognizing 4 bp. Ligation was again performed under dilute conditions to favor intramolecular ligations. Resulting circles were subjected to inverse PCR using primers based on DNA sequences in viewpoints of interest, to co-amplify unknown sequences from interaction partners. Our primers included Ion Torrent A and P1 adapters and barcode sequences. Amplified 4C libraries were size selected and used for high-throughput sequencing.
