# Supplementary material for: 4C-seq characterization of Drosophila BEAF binding regions provides evidence for highly variable long-distance interactions between active chromatin
Source: PLoS One. 2018 Sep 24;13(9):e0203843. doi: 10.1371/journal.pone.0203843 (PMC6152978; doi:10.1371/journal.pone.0203843)
Supplement: S2 Fig — Shown are the Hi-C maps, genome-wide mapping information for various insulator and other chromatin proteins (BEAF, M1BP, ZIPIC, dCTCF, Su(Hw), Ibf1/2, Pita, GAF, Zw5, condensin Cap-H2, CP190, Chromator, cohesion Rad21, Pol II) and histone modifications (H3K36me3, H3K79me3, H3K27me3, H4K16ac, H3K4me1, H3K4me3), binding site motif locations (BEAF, M1BP, motif-6, motif-8, ZIPIC, dCTCF, Su(Hw), Ibf) and gene models around the (A) scs’ viewpoint (CG3281 and aur genes); (B) hts viewpoint (hts and CalpA genes); (C) snf viewpoint (Ckd7 and snf genes); and (D) RpS6 viewpoint (RpS6 and bys genes). See Chorogenome Navigator (http://chorogenome.ie-freiburg.mpg.de/) [52] for details. (PDF) [file pone.0203843.s002.pdf]

Fig S2-A

scs'

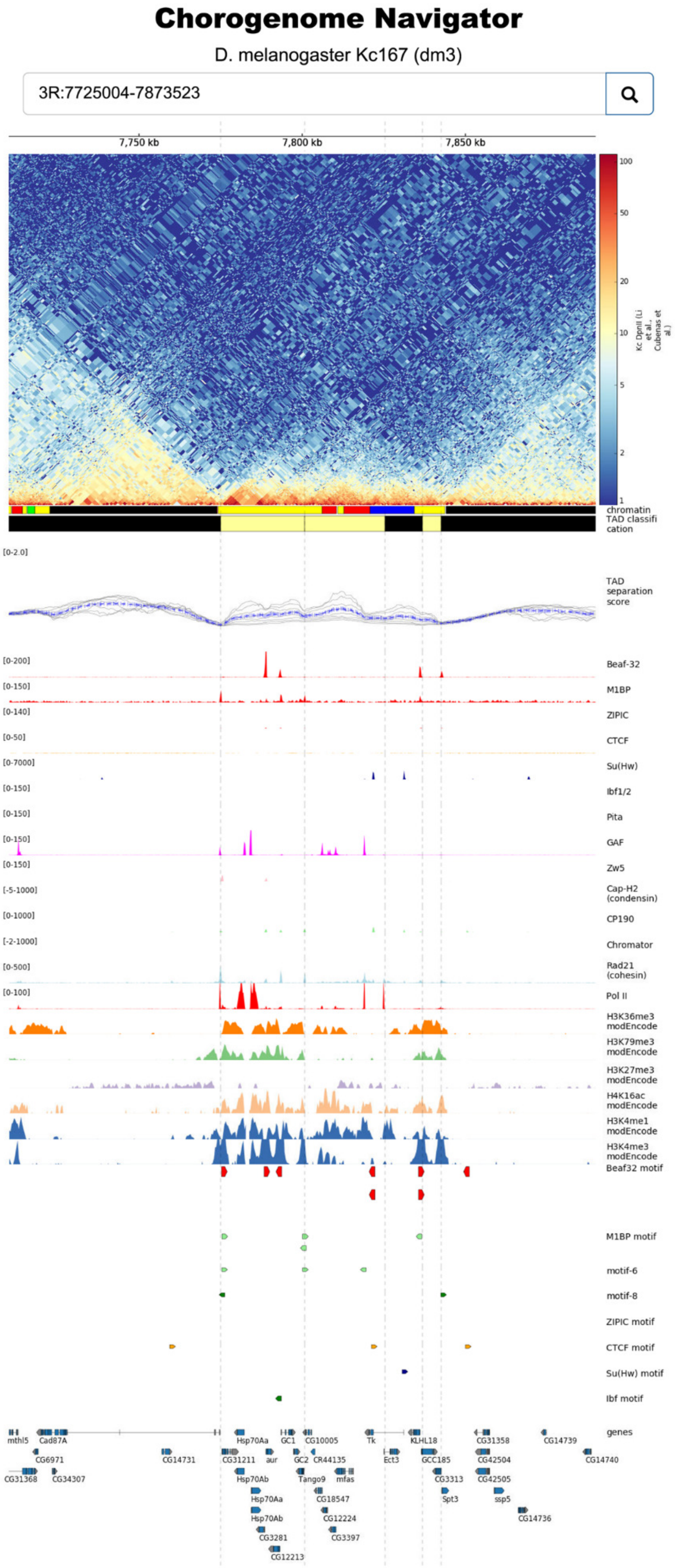
Chorogenome Navigator
D. melanogaster Kc167 (dm3)
3R:7725004-7873523
7,750 kb
7,800 kb
7,850 kb
100
50
20
10
5
2
1
Kc DpnII (Li et al., Cubenas et al.)
chromatin
TAD classification
[0-2.0]
TAD separation score
[0-200]
Beaf-32
[0-150]
M1BP
[0-140]
ZIPIC
[0-50]
CTCF
[0-7000]
Su(Hw)
[0-150]
Ibf1/2
[0-150]
Pita
[0-150]
GAF
[0-150]
Zw5
[-5-1000]
Cap-H2 (condensin)
[0-1000]
CP190
[-2-1000]
Chromator
[0-500]
Rad21 (cohesin)
[0-100]
Pol II
H3K36me3 modEncode
H3K79me3 modEncode
H3K27me3 modEncode
H4K16ac modEncode
H3K4me1 modEncode
H3K4me3 modEncode
Beaf32 motif
M1BP motif
motif-6
motif-8
ZIPIC motif
CTCF motif
Su(Hw) motif
Ibf motif
genes
mthl5
Cad87A
CG6971
CG31368
CG34307
CG14731
Hsp70Aa
CG31211
Hsp70Ab
Hsp70Aa
Hsp70Ab
CG3281
CG12213
aur
GC1
GC2
CG10005
CR44135
Tango9
mfas
CG18547
CG12224
CG3397
Tk
Ect3
KLHL18
GCC185
CG3313
Spt3
CG31358
CG42504
CG42505
ssp5
CG14739
CG14740
CG14736

Fig S2-B

hts

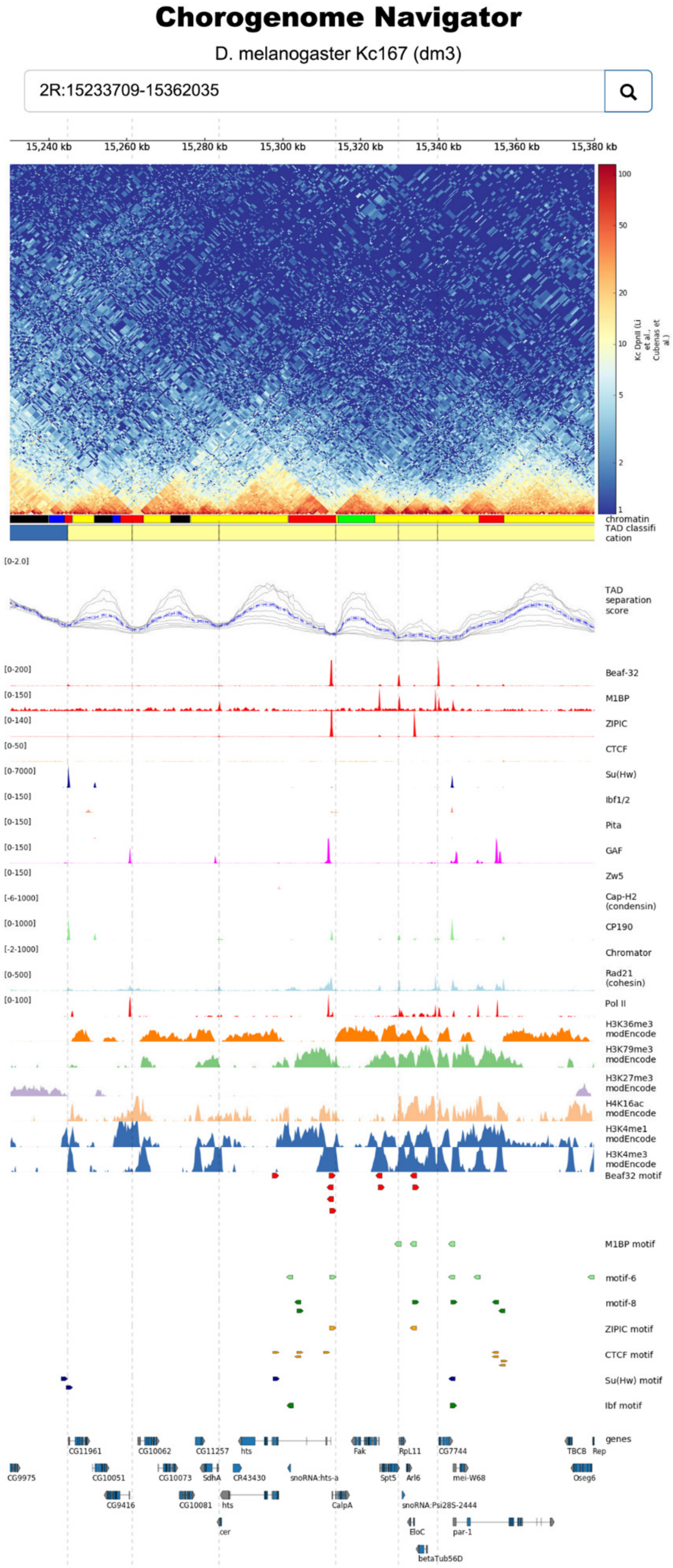
Chorogenome Navigator
D. melanogaster Kc167 (dm3)
2R:15233709-15362035
15,240 kb
15,260 kb
15,280 kb
15,300 kb
15,320 kb
15,340 kb
15,360 kb
15,380 kb
chromatin
TAD classification
TAD separation score
Beaf-32
M1BP
ZIPIC
CTCF
Su(Hw)
Ibf1/2
Pita
GAF
Zw5
Cap-H2 (condensin)
CP190
Chromator
Rad21 (cohesin)
Pol II
H3K36me3 modEncode
H3K79me3 modEncode
H3K27me3 modEncode
H4K16ac modEncode
H3K4me1 modEncode
H3K4me3 modEncode
Beaf32 motif
M1BP motif
motif-6
motif-8
ZIPIC motif
CTCF motif
Su(Hw) motif
Ibf motif
genes

Fig S2-C

snf

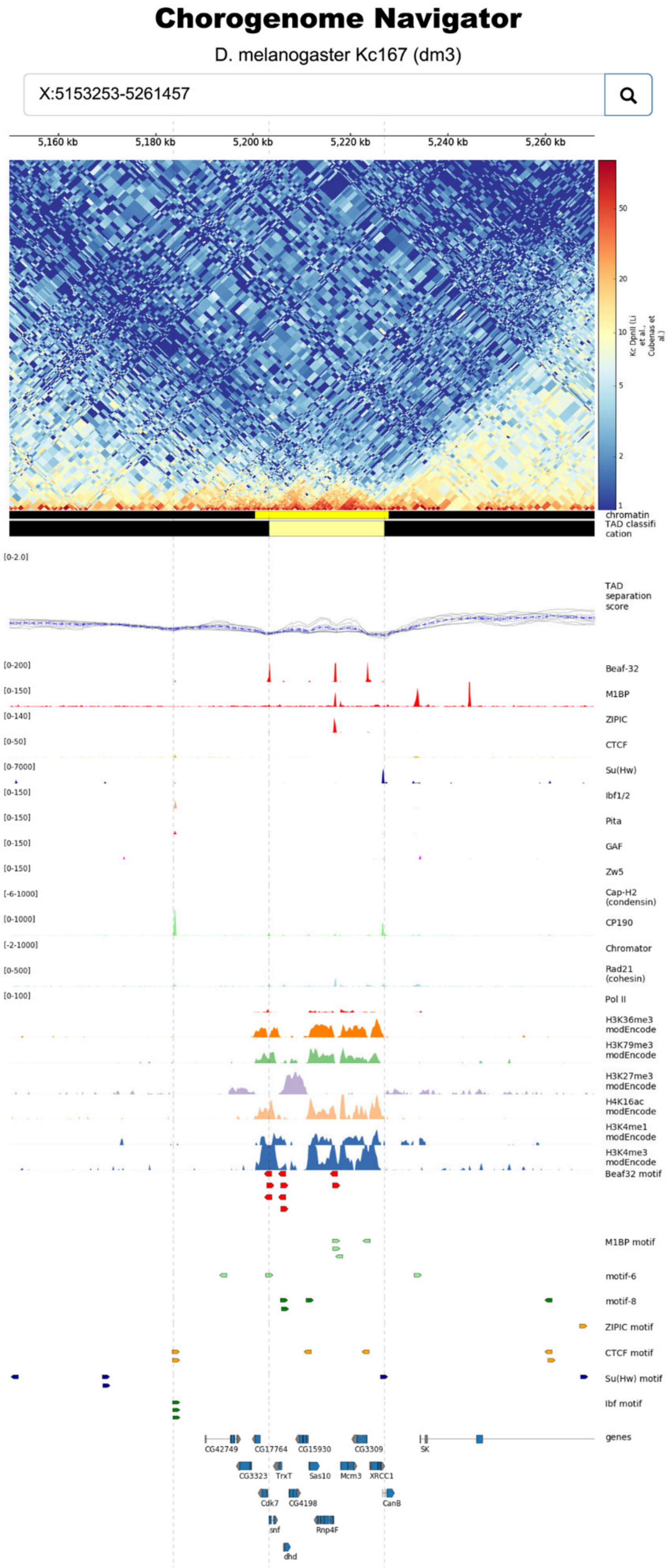
Chorogenome Navigator
D. melanogaster Kc167 (dm3)
X:5153253-5261457
5,160 kb
5,180 kb
5,200 kb
5,220 kb
5,240 kb
5,260 kb
chromatin
TAD classification
TAD separation score
Beaf-32
M1BP
ZIPIC
CTCF
Su(Hw)
Ibf1/2
Pita
GAF
Zw5
Cap-H2 (condensin)
CP190
Chromator
Rad21 (cohesin)
Pol II
H3K36me3 modEncode
H3K79me3 modEncode
H3K27me3 modEncode
H4K16ac modEncode
H3K4me1 modEncode
H3K4me3 modEncode
Beaf32 motif
M1BP motif
motif-6
motif-8
ZIPIC motif
CTCF motif
Su(Hw) motif
Ibf motif
genes
CG42749
CG17764
CG15930
CG3309
SK
CG3323
TrxT
Sas10
Mcm3
XRCC1
Cdk7
CG4198
CanB
snf
Rnp4F
dhd

Fig S2-D

RpS6

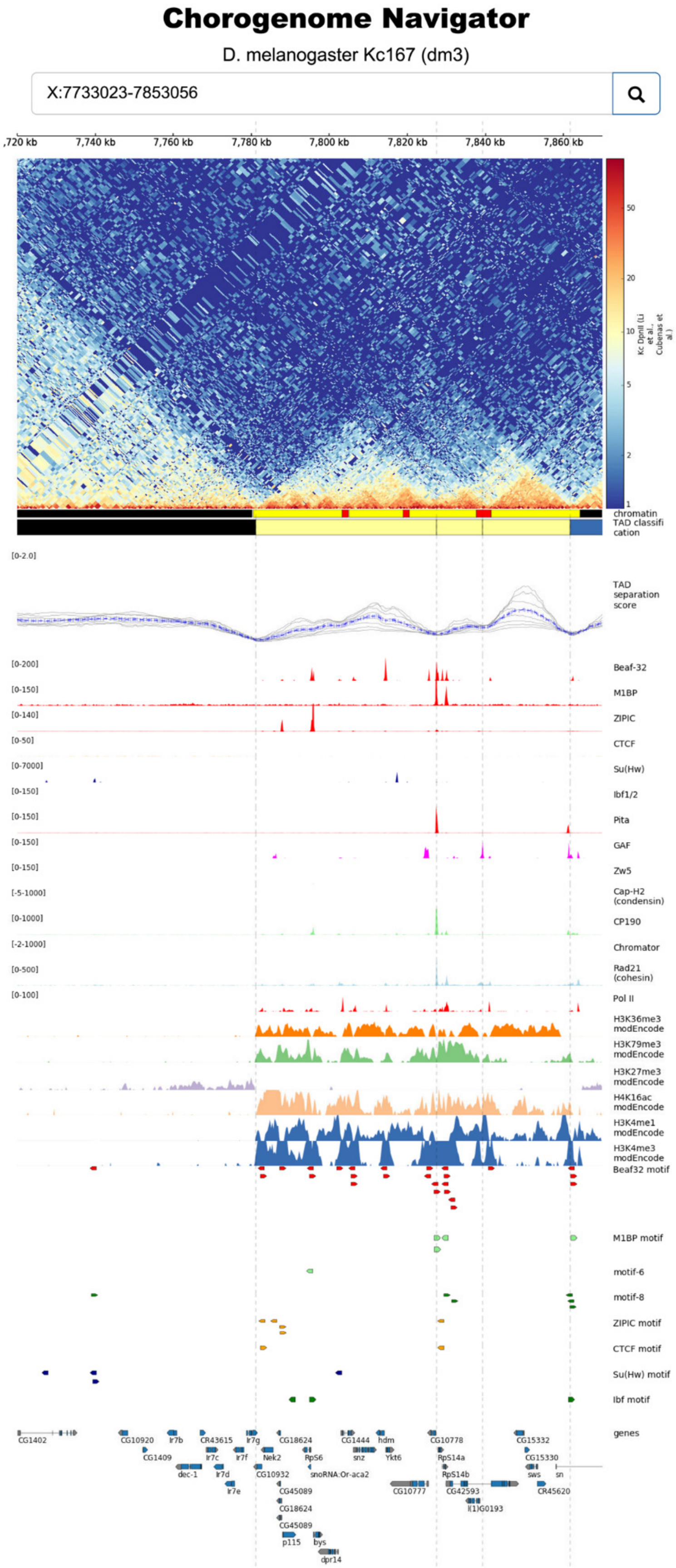
Chorogenome Navigator
D. melanogaster Kc167 (dm3)
X:7733023-7853056
chromatin
TAD classification
TAD separation score
Beaf-32
M1BP
ZIPIC
CTCF
Su(Hw)
Ibf1/2
Pita
GAF
Zw5
Cap-H2 (condensin)
CP190
Chromator
Rad21 (cohesin)
Pol II
H3K36me3 modEncode
H3K79me3 modEncode
H3K27me3 modEncode
H4K16ac modEncode
H3K4me1 modEncode
H3K4me3 modEncode
Beaf32 motif
M1BP motif
motif-6
motif-8
ZIPIC motif
CTCF motif
Su(Hw) motif
Ibf motif
genes

S2 Fig: Chorogenome Navigator snapshots of 1 Mb regions around the viewpoints. Shown are the Hi-C maps, genome-wide mapping information for various insulator and other chromatin proteins (BEAF, M1BP, ZIPIC, dCTCF, Su(Hw), Ibf1/2, Pita, GAF, Zw5, condensin Cap-H2, CP190, Chromator, cohesion Rad21, Pol II) and histone modifications (H3K36me3, H3K79me3, H3K27me3, H4K16ac, H3K4me1, H3K4me3), binding site motif locations (BEAF, M1BP, motif-6, motif-8, ZIPIC, dCTCF, Su(Hw), Ibf) and gene models around the (**A**) scs' viewpoint (*CG3281* and *aur* genes); (**B**) *hts* viewpoint (*hts* and *CalpA* genes); (**C**) *snf* viewpoint (*Ckd7* and *snf* genes); and (**D**) *RpS6* viewpoint (*RpS6* and *bys* genes). See Chorogenome Navigator (http://chorogenome.ie-freiburg.mpg.de/) [52] for details.
