# Supplementary material for: 4C-seq characterization of Drosophila BEAF binding regions provides evidence for highly variable long-distance interactions between active chromatin
Source: PLoS One. 2018 Sep 24;13(9):e0203843. doi: 10.1371/journal.pone.0203843 (PMC6152978; doi:10.1371/journal.pone.0203843)
Supplement: S3 Fig — The highest read densities are around the (A) scs’ viewpoint, (B) hts viewpoint, (C) snf viewpoint, and (D) RpS6 viewpoint for both biological replicates, as is typical of 4C studies. (PDF) [file pone.0203843.s003.pdf]

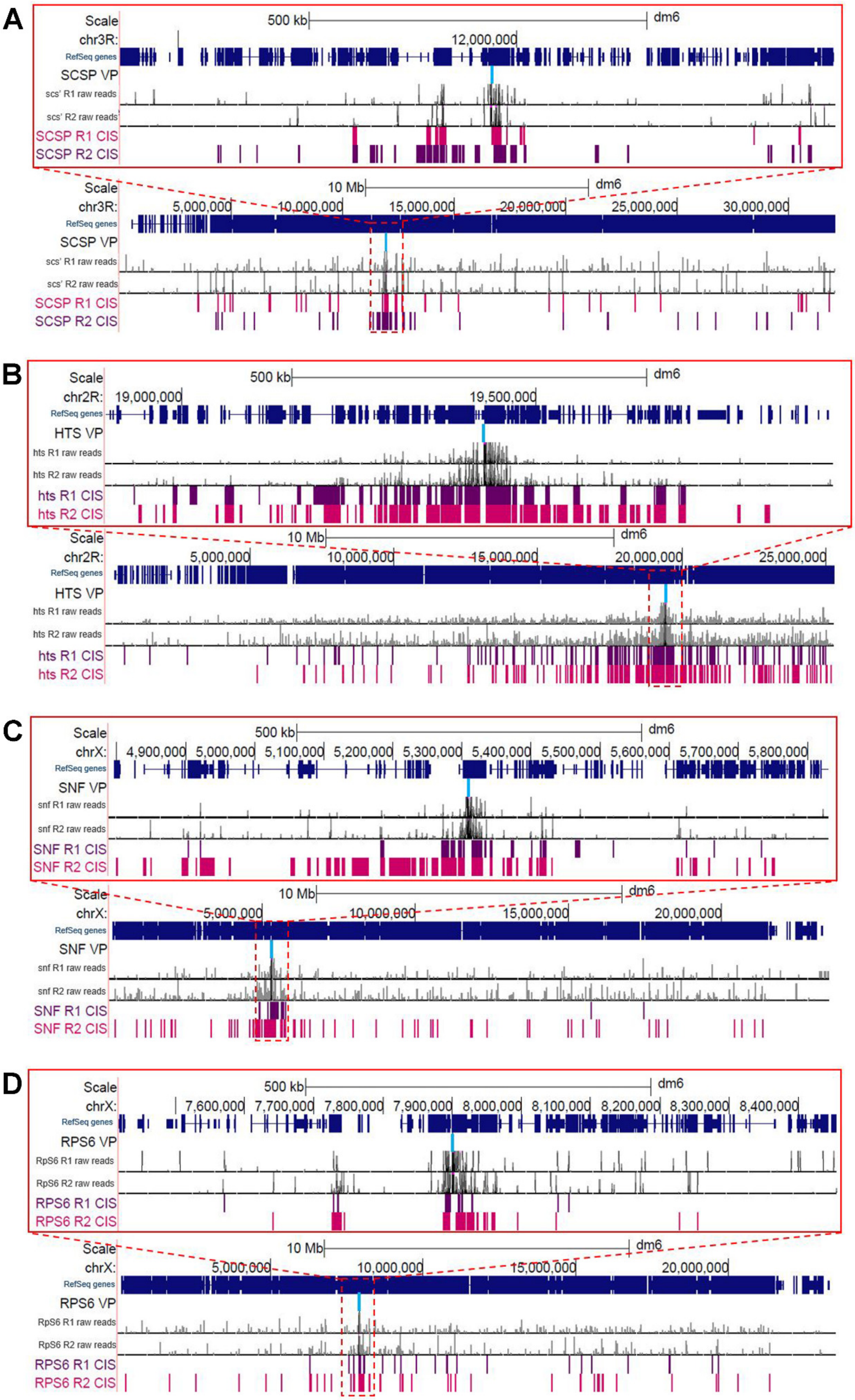
A
Scale
500 kb
dm6
chr3R:
12,000,000
RefSeq genes
SCSP VP
scs' R1 raw reads
scs' R2 raw reads
SCSP R1 CIS
SCSP R2 CIS
Scale
10 Mb
dm6
chr3R:
5,000,000
10,000,000
15,000,000
20,000,000
25,000,000
30,000,000
RefSeq genes
SCSP VP
scs' R1 raw reads
scs' R2 raw reads
SCSP R1 CIS
SCSP R2 CIS
B
Scale
500 kb
dm6
chr2R:
19,000,000
19,500,000
RefSeq genes
HTS VP
hts R1 raw reads
hts R2 raw reads
hts R1 CIS
hts R2 CIS
Scale
10 Mb
dm6
chr2R:
5,000,000
10,000,000
15,000,000
20,000,000
25,000,000
RefSeq genes
HTS VP
hts R1 raw reads
hts R2 raw reads
hts R1 CIS
hts R2 CIS
C
Scale
500 kb
dm6
chrX:
4,900,000
5,000,000
5,100,000
5,200,000
5,300,000
5,400,000
5,500,000
5,600,000
5,700,000
5,800,000
RefSeq genes
SNF VP
snf R1 raw reads
snf R2 raw reads
SNF R1 CIS
SNF R2 CIS
Scale
10 Mb
dm6
chrX:
5,000,000
10,000,000
15,000,000
20,000,000
RefSeq genes
SNF VP
snf R1 raw reads
snf R2 raw reads
SNF R1 CIS
SNF R2 CIS
D
Scale
500 kb
dm6
chrX:
7,600,000
7,700,000
7,800,000
7,900,000
8,000,000
8,100,000
8,200,000
8,300,000
8,400,000
RefSeq genes
RPS6 VP
RpS6 R1 raw reads
RpS6 R2 raw reads
RPS6 R1 CIS
RPS6 R2 CIS
Scale
10 Mb
dm6
chrX:
5,000,000
10,000,000
15,000,000
20,000,000
RefSeq genes
RPS6 VP
RpS6 R1 raw reads
RpS6 R2 raw reads
RPS6 R1 CIS
RPS6 R2 CIS

S3 Fig: 4C interaction and read distributions along the chromosome arms of the viewpoints. Shown are 1 Mb regions centered on the viewpoints (top of each panel) and the entire chromosome arms containing the viewpoints (bottom of each panel), as in Fig 2. The locations of sequence reads and 4C interactions are indicated for replicates R1 and R2. Note that the y-axes for read tracks are set so that reads can be seen for regions with lower counts. Most regions with off-scale read counts are near viewpoints. The highest read densities are around the (**A**) scs' viewpoint, (**B**) *hts* viewpoint, (**C**) *snf* viewpoint, and (**D**) *RpS6* viewpoint for both biological replicates, as is typical of 4C studies.
