# Supplementary material for: 4C-seq characterization of Drosophila BEAF binding regions provides evidence for highly variable long-distance interactions between active chromatin
Source: PLoS One. 2018 Sep 24;13(9):e0203843. doi: 10.1371/journal.pone.0203843 (PMC6152978; doi:10.1371/journal.pone.0203843)
Supplement: S1 Table — (PDF) [file pone.0203843.s004.pdf]

S1 Table.

Primers for checking restriction digestion efficiency while making 4C libraries, and inverse PRC primers for making 4C libraries.

Viewpoint	Chr	Primer name	Primer sequence
**4C restriction digestion check primers**			
scs'	3R	scs'_cut_L5	ATGATGCCGAATCATGGACACACC
		scs'_cut_L3	TCGCTGTTGGGCTGTATCACAAT
		scs'_cut_R5	TGACACAGATGCATAGGCAGATTTGG
		scs'_cut_R3	CGCTTCACAATTCAAACGAACGGC
hts	2R	hts_cut_L5	CTGTAGTTTCGCCACTGTCTATGAAGC
		hts_cut_L3	CAGTGAGTGTGACCGTTGTATTTGTGAC
		hts_cut_R5	GGACAGGAGTTCCTCAATGCAGC
		hts_cut_R3	ATTGTCTCATAGTCCTGCACCTCGG
snf	X	snf_cut_L5	AGCCAATGATATTCTCGTGCTGCAG
		snf_cut_L3	GGAACGCTATGCCAAGCTATCCT
		snf_cut_R5	GACAGGATGGAGATGCTACCCAAC
		snf_cut_R3	TGGGATACTCACTTCTTCTCATCGGTACC
RpS6	X	RpS6_cut_L5	CATCTTTGGAACGTACTCCAATCACA
		RpS6_cut_L3	GGCAACGACAAGCAGGGATT
		RpS6_cut_R5	CACGAGAATGGAGATAATTGCACTGATTC
		RpS6_cut_R3	ATTGTAATCACTACACGTGCTGCAAC
**4C library primers**			
scs'	3R	scs'_inverse_L5	CCTCTCTATGGGCAGTCGGTGATCACAGCTGTTCTGGTTCACAGTCAC
		scs'_inverse_L3	CCATCTCATCCCTGCGTGTCTCCGACTCAGTCTAACGGACGATATGATGCCGAATCATGGACACACC
		scs'_inverse_R5	CCATCTCATCCCTGCGTGTCTCCGACTCAGTTGGAGTGTCGATCGCTTCACAATTCAAACGAACGGC
		scs'_inverse_R3	CCTCTCTATGGGCAGTCGGTGATGATTAACCGGACGATAGACTATCGGAAGATA
hts	2R	hts_inverse_L5	CCTCTCTATGGGCAGTCGGTGATCGTACTACGATTTTGTTAGGGATGGACAAACC
		hts_inverse_L3	CCATCTCATCCCTGCGTGTCTCCGACTCAGTTCGTGATTCGATCAGTGAGTGTGACCGTTGTATTTGTGAC
		hts_inverse_R5	CCATCTCATCCCTGCGTGTCTCCGACTCAGTTCCGATAACGATGGACAGGAGTTCCTCAATGCAGC
		hts_inverse_R3	CCTCTCTATGGGCAGTCGGTGATTGGAGACAAGAATGTCTCGGTTCGC
snf	X	snf_inverse_L5	CCTCTCTATGGGCAGTCGGTGATGCAAAAACAGCGTATTGCTCCTTTATTGC
		snf_inverse_L3	CCATCTCATCCCTGCGTGTCTCCGACTCAGCTAAGGTAACGATGGAACGCTATGCCAAGCTATCCT
		snf_inverse_R5	CCATCTCATCCCTGCGTGTCTCCGACTCAGTAAGGAGAACGATGACAGGATGGAGATGCTACCCAAC
		snf_inverse_R3	CCTCTCTATGGGCAGTCGGTGATGGTGTTTGGCAGTGTTGTTAAACTGC
RpS6	X	RpS6_inverse_L5	CCTCTCTATGGGCAGTCGGTGATGTCGAATTGCAGCGGAGATAATATCATCCA
		RpS6_inverse_L3	CCATCTCATCCCTGCGTGTCTCCGACTCAGTGAGCGGAACGATGGCGGCAACGACAAGCAGG
		RpS6_inverse_R5	CCATCTCATCCCTGCGTGTCTCCGACTCAGCTGACCGAACGATACACGAGAATGGAGATAATTGCACTGATTC
		RpS6_inverse_R3	CCTCTCTATGGGCAGTCGGTGATCGAGTGTTATTTTCGTACTTATTTGGCATAATTC
