# Supplementary material for: 4C-seq characterization of Drosophila BEAF binding regions provides evidence for highly variable long-distance interactions between active chromatin
Source: PLoS One. 2018 Sep 24;13(9):e0203843. doi: 10.1371/journal.pone.0203843 (PMC6152978; doi:10.1371/journal.pone.0203843)
Supplement: S2 Table — (PDF) [file pone.0203843.s005.pdf]

S2 Table.
Virtual 4C analysis of viewpoints using the indicated Hi-C data.
See Table 1 for details.

Viewpoint	Number of *cis*-interactions	Common *cis*-interactions (%)	Common interactions that are near-*cis* (%)	Unique interactions that are near-*cis* (%)	Number of *trans*-interactions	Common *trans*-interactions (%)
Kc cells: El-Sharnouby et al. 2017 PLoS One 12:e0172725						
scs'_R1	337	32	95	34	87	0
scs'_R2	333	33	95	29	112	0
hts_R1	427	30	84	19	317	17
hts_R2	421	31	84	21	308	16
snf_R1	367	39	96	40	101	0
snf_R2	396	36	96	35	137	0
RpS6_R1	364	27	87	30	127	1
RpS6_R2	305	32	87	34	115	1
S2 cells: Szabo et al. 2018 Sci. Adv. 4:eaar8082						
scs'_R1	842	46	77	14	182	4
scs'_R2	1174	33	77	24	464	2
hts_R1	1446	49	42	11	206	2
hts_R2	2094	34	42	20	793	1
snf_R1	771	39	70	23	59	0
snf_R2	961	32	70	34	282	0
RpS6_R1	401	39	83	24	28	0
RpS6_R2	596	26	83	39	187	0
S2 cells: Wang et al. 2018 Nat. Commun. 9:188						
scs'_R1	1097	27	79	17	552	3
scs'_R2	533	56	79	3	194	8
hts_R1	1001	20	65	12	407	2
hts_R2	487	41	65	12	151	6
snf_R1	715	18	71	19	197	2
snf_R2	297	44	71	20	81	5
RpS6_R1	790	22	68	16	225	2
RpS6_R2	420	41	68	15	94	5

Cubenas-Potts et al. 2017 Nucleic Acids Res. 45:1714-30 (Kc cells) was also analyzed but gave only near-*cis* interactions and is not included in this table.
