# Supplementary material for: 4C-seq characterization of Drosophila BEAF binding regions provides evidence for highly variable long-distance interactions between active chromatin
Source: PLoS One. 2018 Sep 24;13(9):e0203843. doi: 10.1371/journal.pone.0203843 (PMC6152978; doi:10.1371/journal.pone.0203843)
Supplement: S3 Table — (PDF) [file pone.0203843.s006.pdf]

S3A Table.

Primers for 3C validation of 4C interactions, and 3C results.

Primer name indicates if the 4C cis-interaction was unique to one or common to both 4C replicates.

*: 3C positive control; previously reported interaction between scs' and scs.

**: Region selected for FISH analysis.

Viewpoint	Primer name	Chr	Primer sequence	Distance from viewpoint	3C result	
					Rep1	Rep2
scs'	scs'_VP_5	3R	GTCACGATATTCTTCAACCAACCGATAGTATCGC	viewpoint		
	scs'_scs_common_cis_3*	3R	ATCGTTTACGGTAGCCATTCTCACGT	~ 11 kb	+	+
	scs'_common_cis1_3	3R	TAATCTCCACCTGCCATTTG	~ 49 kb	+	+
	scs'_common_cis2_3	3R	CGAACGACACTCGCAATTA	~ 201 kb	-	-
	scs'_common_cis3_3**	3R	CAACTCTCGCTCTCGTTTC	~ 203 kb	+	-
	scs'_common_cis4_3	3R	CAGGAGAAGAAGGTGGTTTAAG	~ 204 kb	-	-
	scs'_common_cis5_3	3R	ACAGTGAACTCGTCGTCA	~ 457 kb	-	-
	scs'_common_cis6_3	3R	ACGTCCACCAGTCCCTTCACAAT	~ 527 kb	+	+
	scs'_common_cis7_3**	3R	CGAACTCCATTGTGAACAAGCTGATGG	~ 748 kb	+	+
	scs'_common_cis8_3	3R	CCTCAAATGAGATGTAGCCGT	~ 930 kb	+	-
	scs'_unique_cis1_R1	3R	GCTGCTGCTAACTCAATTATAC	~ 1.28 Mb	-	+
	scs'_unique_cis1_R2	3R	ATTGCCATTGCCGTTGTGCATCATATC	~ 2.46 Mb	-	+
	scs'_unique_cis2_R2	3R	TGTACTCACCGAAGCTTTATTC	~ 3.84 Mb	-	-
	scs'_unique_cis2_R1	3R	GCTTGTCACTCATCCATTCTATC	~ 17.1 Mb	-	+
hts	hts_VP_5	2R	ATCTGAGGCGGCACCTTACACT	viewpoint		
	hts_common_cis1_3	2R	TAAGCTCCAGAGAACGCTTTAGTGC	~ 62 kb	+	-
	hts_common_cis2_3	2R	TGAATTTCCCAATGCCCAAATCAACAGGT	~ 67 kb	+	+
	hts_common_cis3_3	2R	GGGTTTCCTTATTGCAAGGG	~ 115 kb	+	-
	hts_common_cis4_3**	2R	TCACAATGGCCTTCACATC	~ 850 kb	+	+
	hts_common_cis5_3	2R	GCGCCCACAAAGAACTTA	~ 896 kb	+	+
	hts_common_cis6_3	2R	CTGTGTCTCAGTGCATAGAAG	~ 1.29 Mb	+	-
	hts_common_cis7_3	2R	CCTATTAACGCGGTCATATCCT	~ 1.39 Mb	-	-
	hts_common_cis8_3	2R	CAATGTCGGCGTGTATGT	~ 1.69 Mb	-	-
	hts_common_cis9_3	2R	CTTAACCGGCTTTGCCTT	~ 1.73 Mb	-	-
	hts_unique_cis1_R2	2R	AATCCGAAGAACTTTGGCAATA	~ 3.45 Mb	-	-
	hts_common_cis10_3	2R	GCACTCTTGCCGTAGGA	~ 4.49 Mb	-	-
	hts_common_cis11_3	2R	CCGCATTCGTGTTGTATTTG	~ 4.85 Mb	-	-
	hts_unique_cis1_R1	2R	CCGTGGTTCTCGGTGATAACAGCATAG	~ 5.47 Mb	-	-
	hts_unique_cis2_R2	2R	CTCGTACTTGCTATCCACGTACTCCAGTA	~ 11.2 Mb	-	+
	hts_unique_cis2_R1	2R	GTTATGGTGATTATTGGTAGATTCG	~ 12.8 Mb	-	+
	hts_trans1_3	X	GGAAAGACCGCGTTATATCTG	trans	+	-
	hts_trans2_3	3L	GTTGATAGTAGCCCGGTTGA	trans	-	-
	hts_trans3_3	2L	TGCACTCCTCAGAACATAAAC	trans	+	-
snf	snf_VP_5	X	CACCGGTGCGATAACATATCGATAGACC	viewpoint		
	snf_common_cis1_3	X	AATGGTGGACGATTGGG	~ 7 kb	+	-
	snf_common_cis2_3	X	AACGGTTGAAGCATGGAC	~ 14 kb	+	+
	snf_common_cis3_3	X	CTAAGTGTAGCACAGCCACA	~ 105 kb	+	-
	snf_common_cis4_3	X	ATGCCGAGCATAAGTGCGTCTGTG	~ 112 kb	+	+
	snf_common_cis5_3	X	GAAATCACCACCGACGT	~ 231 kb	-	-
	snf_common_cis6_3	X	GCAACACAAGCAACATACAACTGTGGT	~ 370 kb	+	+
	snf_common_cis7_3**	X	GTAATACGGCTCCTGCATGA	~ 405 kb	+	-
	snf_unique_cis1_R2	X	GCTATCCATTAACCAATACTCCCGAGCGA	~ 1.21 Mb	-	+
	snf_common_cis8_3	X	GCAGATTATGTTTGGCTTGTG	~ 4.75 Mb	-	-
	snf_unique_cis2_R2	X	ATGTACATACATCCATCCATTCG	~ 6.51 Mb	-	+
	snf_common_cis9_3	X	CCAATAAGCGCATTCTACACA	~ 9.49 Mb	-	-
	snf_unique_cis1_R1	X	GTGCCTTCTCGAATCCATCGGTCA	~ 10.4 Mb	-	-
	snf_unique_cis2_R1	X	ATCGAATCGTTTCATTGTTGTG	~ 12.1 Mb	-	+
	snf_trans1_3	3L	TTCGCTCCACTTGAACTTGTGCT	trans	+	+
	snf_trans2_3	2L	ACTGAGTCTAGGAAATATGAAACC	trans	-	-
	snf_trans3_3	2R	GCTGACCCATGAAGCTCCACTT	trans	-	-
RpS6	RpS6_VP_5	X	GACCGATAGGAGGAAGTAGAGATGACAGAT	viewpoint		
	RpS6_common_cis1_3	X	ATCCCGTCGATCCTCCAGGA	~ 7 kb	+	+
	RpS6_common_cis2_3	X	ACCGCTAAACCAATGTCCAAGTCATCGT	~ 9 kb	+	+
	RpS6_common_cis3_3	X	ATACGATCGGAAGAGCGATG	~ 10 kb	+	+
	RpS6_common_cis4_3	X	GCTATACATTACTTATCTGGAAAGG	~ 13 kb	+	+
	RpS6_common_cis5_3	X	GTGTTTAGGATCTCGACCAGTG	~ 14 kb	+	+
	RpS6_common_cis6_3	X	GTATAAACAGCACCGGCACTGCT	~ 142 kb	+	-
	RpS6_common_cis7_3	X	CACAAATCTATGCATTACATCCC	~ 167 kb	+	-
	RpS6_common_cis8_3	X	CCCAAATCCTGATGATCCTTATC	~ 172 kb	-	-
	RpS6_common_cis9_3	X	AAGTCCGTTGCTTCTTCTC	~ 1.61 Mb	-	-
	RpS6_common_cis10_3**	X	CGATAAACAGAGCACCAAAGA	~ 3.05 Mb	+	+
	RpS6_unique_cis1_R2	X	GCACAACAAAGGACTAGAAGTA	~ 3.05 Mb	-	+
	RpS6_unique_cis1_R1	X	CAATCGGCATTCTATTGTTTGA	~ 3.20 Mb	-	+
	RpS6_common_cis11_3	X	TAGTGTCAGCACTTGGCTGTCACA	~ 5.05 Mb	-	-
	RpS6_common_cis12_3	X	GTAAAATCTTGTAGCCAGAACAACAACAGCAG	~ 6.15 Mb	+	+
	RpS6_unique_cis2_R1	X	CTACTATTGTCCGCACCACTGTCGAT	~ 7.58 Mb	-	+
	RpS6_unique_cis2_R2	X	CTCGTCGATCACCTCCAGCAGATA	~ 11.8 Mb	-	+
	RpS6_trans1_3	2L	CACTCTCAGGTTATAAAATCCACTCAAGGCAAA	trans	-	-
	RpS6_trans2_3	2L	TACTTGGCTGACTCCGG	trans	+	-
	RpS6_trans3_3	3L	GATCATTAGCACTAGCAATTAGCTGCGC	trans	+	+

S3B Table: 3C cross-check.
Primers for 3C validation of 4C interactions, and 3C results.
Primer name indicates if the 4C cis-interaction was unique to one or common to both 4C replicates.
*: 3C positive control; previously reported interaction between scs' and scs.
**: Region selected for FISH analysis.

3C viewpoint cross-check: viewpoint, primer name, chr, sequence, original viewpoint interaction, and results						
**Viewpoint**	**Primer name**	**Chr**	**Primer sequence**	**Distance from viewpoint**	**3C result Rep1**	**Rep2**
scs'	hts_common_cis1_3	2R	TAAGCTCCAGAGAACGCTTTAGTGC	hts	-	-
	hts_common_cis2_3	2R	TGAATTTCCCAATGCCCAAATCAACAGGT	hts	-	-
	snf_common_cis4_3	X	ATGCCGAGCATAAGTGCGTCTGTG	snf	-	-
	snf_common_cis6_3	X	GCAACACAAGCAACATACAACTGTGGT	snf	-	-
	snf_trans3_3	2R	GCTGACCCATGAAGCTCCACTT	snf	-	-
	RpS6_common_cis1_3	X	ATCCCGTCGATCCTCCAGGA	RpS6	-	-
	RpS6_common_cis2_3	X	ACCGCTAAACCAATGTCCAAGTCATCGT	RpS6	-	-
	RpS6_trans3_3	3L	GATCATTAGCACTAGCAATTAGCTGCGC	RpS6	-	-
hts	scs'_common_cis6_3	3R	ACGTCCACCAGTCCCTTCACAAT	scs'	-	-
	scs'_common_cis7_3	3R	CGAACTCCATTGTGAACAAGCTGATGG	scs'	-	-
	snf_common_cis4_3	X	ATGCCGAGCATAAGTGCGTCTGTG	snf	-	-
	snf_common_cis6_3	X	GCAACACAAGCAACATACAACTGTGGT	snf	-	-
	snf_trans3_3	2R	GCTGACCCATGAAGCTCCACTT	snf	-	-
	RpS6_common_cis1_3	X	ATCCCGTCGATCCTCCAGGA	RpS6	-	-
	RpS6_common_cis2_3	X	ACCGCTAAACCAATGTCCAAGTCATCGT	RpS6	-	-
	RpS6_trans3_3	3L	GTAAAATCTTGTAGCCAGAACAACAACAGCAG	RpS6	-	-
snf	scs'_common_cis6_3	3R	ACGTCCACCAGTCCCTTCACAAT	scs'	-	-
	scs'_common_cis7_3	3R	CGAACTCCATTGTGAACAAGCTGATGG	scs'	-	-
	hts_common_cis1_3	2R	TAAGCTCCAGAGAACGCTTTAGTGC	hts	-	-
	hts_common_cis2_3	2R	TGAATTTCCCAATGCCCAAATCAACAGGT	hts	-	-
	RpS6_common_cis1_3	X	ATCCCGTCGATCCTCCAGGA	RpS6	-	-
	RpS6_common_cis2_3	X	ACCGCTAAACCAATGTCCAAGTCATCGT	RpS6	-	-
	RpS6_trans3_3	3L	GTAAAATCTTGTAGCCAGAACAACAACAGCAG	RpS6	-	-
RpS6	scs'_common_cis6_3	3R	ACGTCCACCAGTCCCTTCACAAT	scs'	-	-
	scs'_common_cis7_3	3R	CGAACTCCATTGTGAACAAGCTGATGG	scs'	-	-
	hts_common_cis1_3	2R	TAAGCTCCAGAGAACGCTTTAGTGC	hts	-	-
	hts_common_cis2_3	2R	TGAATTTCCCAATGCCCAAATCAACAGGT	hts	-	-
	snf_common_cis4_3	X	ATGCCGAGCATAAGTGCGTCTGTG	snf	-	-
	snf_common_cis6_3	X	GCAACACAAGCAACATACAACTGTGGT	snf	-	-
	snf_trans3_3	2R	GCTGACCCATGAAGCTCCACTT	snf	-	-
