# Supplementary material for: 4C-seq characterization of Drosophila BEAF binding regions provides evidence for highly variable long-distance interactions between active chromatin
Source: PLoS One. 2018 Sep 24;13(9):e0203843. doi: 10.1371/journal.pone.0203843 (PMC6152978; doi:10.1371/journal.pone.0203843)
Supplement: S4 Table — (PDF) [file pone.0203843.s007.pdf]

S4 Table.
Primers for preparing FISH probes.

Viewpoint	Primer name	Chr	Start	End	Primer sequence	Distance from the viewpoint
scs'_viewpoint	5' Primer_1	3R	11959448	11968943	GATCTTAAGGTCGTTGGACTG	–
	3' Primer_1				TCGTGACTTTGTAGTGTTAGAA	
	5' Primer_2				TGTTCTGTTAGTTGTCGAGTG	
	3' Primer_2				AACCCATTTCGCTAGAGATTAG	
	5' Primer_3				ACTGCTAATTCGGGACTAATAA	
	3' Primer_3				CAACTGCAACTATGTACCTAAAG	
	5' Primer_4				AATCTTGTGCTCACCATCG	
	3' Primer_4				GCGGCACCTAACAGTAAAT	
*scs'*_cis_750kb	5' Primer_1	3R	12707806	12714806	CGACTTTCGAACAGCTCATA	~ 750 kbp
	3' Primer_1				GGTAAACGAAACACCCATAAAC	
	5' Primer_2				CTGCAACACATTGAAGCAATA	
	3' Primer_2				GGCGAACTCCATTGTGAA	
	5' Primer_3				CCACATGAAGGCTCTCAAA	
	3' Primer_3				TCAGGCTTACACTTAACTACAC	
	5' Primer_4				TGCGCTGAAATCGCTAAC	
	3' Primer_4				GGGCAAACTGTCCCTGTATA	
*scs'*_cis_200kb	5' Primer_1	3R	11756973	11763773	TAGCCGGGCGACTATTTA	~ 200 kbp
	3' Primer_1				CCGCAAATTGGCAACTTATT	
	5' Primer_2				TAAGAGCTGTGACGGCAGAA	
	3' Primer_2				GCAGGCTTCTAACTACGTACATATC	
	5' Primer_3				TAATTGCGAGTGTCGTTCG	
	3' Primer_3				AGAGACGGCACTAGTATCTG	
	5' Primer_5				CTTGACAAGCAGATTAGAAATTAGG	
	3' Primer_5				TGAACGATTCCAACCAGAAG	
*hts*_viewpoint	5' Primer_1	2R	19423624	19426142	ACCAACGAGTCACGTCTAA	–
	3' Primer_1				CGGTGAGTATTCTTGCATCTAC	
	5' Primer_2				CACTCACTGTATGTGTTAGAGG	
	3' Primer_2				TCGTCATAGGCACTTGATATTT	
	5' Primer_3				GGGTTTCGCCGTAGAATTA	
	3' Primer_3				TTCTGGCTGCGGAAATAG	
	5' Primer_4				GAGAACTATGCTGGTATCTTCC	
	3' Primer_4				CAGGAATGTGTAGAGGGTAAAG	
*hts*_cis_850kb	5' Primer_1	2R	20271926	20278427	TGCAAATATCGAGTAACTAAGAGG	~ 850 kb
	3' Primer_1				CGTTAATCAAACGCAAGTACAG	
	5' Primer_2				TGATCAGAGACCACCAAGTA	
	3' Primer_2				TTCCAGTTCATCCTCCGT	
	5' Primer_3				GTCAGTTCATGGAGGGATTG	
	3' Primer_3				TGCCAACATGATCCAGAAG	
	5' Primer_4				GGATGGCGCTGAATAAAGTAGTAAAG	
	3' Primer_4				AATCCAGACAGAGAGCGCAA	
*snf*_viewpoint	5' Primer_5	X	5303600	5312600	GATTCTTACGCAACTGACGA	–
	3' Primer_5				CAATCAGATACGCCAGTTAGAT	
	5' Primer_1				AGCCATCTAGTTGATCCTAGT	
	3' Primer_1				GTGCACGCTACCGTATTT	
	5' Primer_2				TCGCTCGCTTTCAATCAC	
	3' Primer_2				AAGGAGCAATACGCTGTTT	
	5' Primer_3				GGTGCGATAACATATCGATAGAC	
	3' Primer_3				CGAGGACTTGTGGGACTT	
*snf*_cis_404kb	5' Primer_1	X	4900951	4907451	GGCCAGAAAGCAAATATAGGG	~ 404 kbp
	3' Primer_1				CACCACTACCCTGGTTAGAA	
	5' Primer_2				CTTGCAACCAGATTGATTTATGC	
	3' Primer_2				AACCACCAACCGATCCAA	
	5' Primer_3				TGCACGGCCTGAGTATTT	
	3' Primer_3				TGGCGAACAACTTAAGTGAATA	
	5' Primer_5				GCCGCATGAGTGGAATTT	
	3' Primer_5				CCATCAACTGCCAAGATCAG	
*RpS6*_viewpoint	5' Primer_1	X	7897240	7906240	CGCGGTGTTTACTTCTTGT	–
	3' Primer_1				TGTCATCTCTACTTCCTCCTATC	
	5' Primer_2				GTGCTCACGGCGATATTTA	
	3' Primer_2				AGTGCAGGACGGGAATA	
	5' Primer_3				GCTGAAGATCTGTGAGATGG	
	3' Primer_3				CGATCAAGAACAAGAAGCAAAT	
	5' Primer_4				GTATTTACCCATTGGTCCGA	
	3' Primer_4				TGATATCATTTGCAGTGTCAAG	
*RpS6*_cis_3054kb	5' Primer_1	X	4841173	4853293	CACACACACAGAGAGTGATAAT	~ 3 million bp
	3' Primer_1				TTACACACTGTTGGCACTTTA	
	5' Primer_2				CTGACTGCAAGCCATACATC	
	3' Primer_2				GGCACAGCCTATTGTCTTAAA	
	5' Primer_3				TATTGTATTTGCCCGCCTTAC	
	3' Primer_3				CGATAAACAGAGCACCAAAGA	
	5' Primer_4				TGGAGAGCATCGTCTTATAAAT	
	3' Primer_4				CACAAACAAACGAGAGACATAG	
	5' Primer_5				GTGGAGAAGCACTAACACAC	
	3' Primer_5				AACTGATTTCGCATTGAATCAC	
	5' Primer_6				TGACAGTGATTTCCTCCAATC	
	3' Primer_6				CTGACTCAATCGTCCATCTTT	
