# Supplementary material for: 4C-seq characterization of Drosophila BEAF binding regions provides evidence for highly variable long-distance interactions between active chromatin
Source: PLoS One. 2018 Sep 24;13(9):e0203843. doi: 10.1371/journal.pone.0203843 (PMC6152978; doi:10.1371/journal.pone.0203843)
Supplement: S5 Table — (PDF) [file pone.0203843.s008.pdf]

S5 Table.
For each viewpoint replicate (R1, R2), the total number of reads after trimming off viewpoint sequences,
the % that uniquely aligned to the Drosophila genome R6,
the number of uniquely aligned reads that passed the filter,
the % of filtered reads that were on the same chromosome arm as the viewpoint (% cis reads),
% cis reads in significant 4C interactions,
and the % of trans reads in signicant 4C interactions are shown.

Viewpoint	Trimmed reads	% uniquely aligned	Filtered, uniquely aligned reads	% *cis* reads	% *cis* reads in significant 4C interactions	% *trans* reads in significant 4C interactions
scs'_R1	53184162	64	32388871	39	27	15
scs'_R2	15459739	66	9342247	31	42	56
hts_R1	2977423	70	1763844	29	49	55
hts_R2	6810300	77	5099405	40	56	48
snf_R1	5789427	84	1989581	29	30	13
snf_R2	4504081	75	2677886	31	43	27
RpS6_R1	3253656	73	1420646	22	14	21
RpS6_R2	16325192	85	2333122	31	42	23
