# Supplementary material for: 4C-seq characterization of Drosophila BEAF binding regions provides evidence for highly variable long-distance interactions between active chromatin
Source: PLoS One. 2018 Sep 24;13(9):e0203843. doi: 10.1371/journal.pone.0203843 (PMC6152978; doi:10.1371/journal.pone.0203843)
Supplement: S6 Table — (PDF) [file pone.0203843.s009.pdf]

S6 Table.

FISH nuclei count data, corresponding to Figure 4.

KOB: *BEAF[AB-KO]* , brain; KOW: *BEAF[AB-KO]* , wing disc;

WTB: BEAF wild type, brain; WTW: BEAF wild type, wing disc.

Sample name	Nuclei with colocalization	Total nuclei counted	% colocalization
KOB_scs'_cis_750kb	96	250	38
KOW_scs'_cis_750kb	111	408	27
WTB_scs'_cis_750kb	92	396	23
WTW_scs'_cis_750kb	95	282	34
KOB_scs'_cis_200kb	93	261	36
KOW_scs'_cis_200kb	45	240	19
WTB_scs'_cis_200kb	81	345	23
WTW_scs'_cis_200kb	93	525	18
KOB_hts_cis_850kb	31	135	23
KOW_hts_cis_850kb	66	179	37
WTB_hts_cis_850kb	41	131	31
WTW_hts_cis_850kb	37	136	27
KOB_hts_trans_neg	22	382	6
KOW_hts_trans_neg	16	199	8
WTB_hts_trans_neg	17	187	9
WTW_hts_trans_neg	6	369	2
KOB_snf_cis_404kb	70	332	21
KOW_snf_cis_404kb	48	296	16
WTB_snf_cis_404kb	91	228	40
WTW_snf_cis_404kb	74	298	25
KOB_snf_cis_462kb_neg	8	352	2
KOW_snf_cis_462kb_neg	7	311	2
WTB_snf_cis_462kb_neg	13	161	8
WTW_snf_cis_462kb_neg	21	305	7
KOB_RpS6_cis_3054kb	30	217	14
KOW_RpS6_cis_3054kb	32	158	20
WTB_RpS6_cis_3054kb	37	255	15
WTW_RpS6_cis_3054kb	58	259	22
KOB_RpS6_cis_2997kb_neg	14	226	6
KOW_RpS6_cis_2997kb_neg	16	226	7
WTB_RpS6_cis_2997kb_neg	8	317	3
WTW_RpS6_cis_2997kb_neg	17	293	6
